# Hypoxia increases RCC stem cell phenotype via altering the androgen receptor (AR)-lncTCFL5-2-YBX1-SOX2 signaling axis

**DOI:** 10.1186/s13578-022-00912-5

**Published:** 2022-11-17

**Authors:** Changcheng Guo, Yin Sun, Wei Zhai, Xudong Yao, Dongkui Gong, Bosen You, Chi-Ping Huang, Junhua Zheng, Chawnshang Chang

**Affiliations:** 1 Department of Urology, Shanghai Tenth People’s Hospital, Tongji University, Shanghai, 200072 China; 2 George Whipple Lab for Cancer Research, Departments of Pathology, Urology, Radiation Oncology and The Wilmot Cancer Institute, University of Rochester Medical Center, Rochester, NY 14642 USA; 3 Department of Urology, Renji Hospital, Shanghai Jiaotong University School of Medicine, Shanghai, 400062 China; 4 Department of Urology, China Medical University/Hospital, Taichung, 404 Taiwan

**Keywords:** Hypoxia, Renal cell carcinoma, Cancer stem cells, Androgen receptor, lncTCFL5-2

## Abstract

**Background:**

Early studies indicated that the androgen receptor (AR) could promote renal cell carcinoma (RCC) development and metastasis, but its linkage to RCC progression under hypoxia, remains unclear.

**Results:**

Here we found AR expression in RCC cells decreased in response to hypoxia, which might then lead to increase the cancer stem cells (CSC) phenotype through the lncTCFL5-2-modulated YBX1/SOX2 signals. The consequences of such hypoxia-modulated AR/lncTCFL5-2/YBX1/SOX2 signals ablity to alter the CSC phenotype might render RCC cells more resistant to targeted therapy with Sunitinib. Mechanism dissection revealed that AR might alter the lncTCFL5-2/YBX1/SOX2 signaling through transcriptional suppression of the lncTCFL5-2 expression via the AR-response-elements (AREs) on the lncTCFL5-2 promoter. The lncTCFL5-2 interacts with YBX1 to increase its stability, which in turn increases SOX2 expression at a transcriptional level via the YBX1-response-elements (YBX1Es) on the SOX2 promoter. The in vivo mouse model with orthotopic xenografts of RCC cells also validates the in vitro data, and a human RCC sample survey demonstrated the clinical significance of the AR/lncTCFL5-2/YBX1/SOX2 signaling axis for the RCC prognosis, likely as a result of regulating CSC phenotypes.

**Conclusions:**

Together, these findings suggest that hypoxia may increase the RCC CSC phenotype via altering the AR/lncTCFL5-2/YBX1/SOX2 signaling axis and a potential therapy to target this newly identified signal perhaps may help improve the targeted therapy with Sunitinib to better suppress RCC progression.

**Supplementary Information:**

The online version contains supplementary material available at 10.1186/s13578-022-00912-5.

## Introduction

Renal cell carcinoma (RCC) is the ninth most common malignant tumor representing approximately 2–3% of all adult malignancies [1]. Until recently, there was a worldwide annual increase in incidence of about 2%. Approximately 84,400 new RCC cases were diagnosed resulting in more than 34,700 kidney cancer-related deaths within the European Union in 2017 [2]. Although there is a higher incidence of detection of small renal masses through improved diagnosis, approximately one-third of patients will develop metastatic lesions during the course of the disease [3]. Therapeutic targeting of critical biological pathways, including those involving the vascular endothelial growth factor (VEGF) and the mammalian target of rapamycin (mTOR), has produced robust clinical effects and revolutionized the treatment of metastatic RCC [4]. However, some patients are inherently resistant to these approaches and most, if not all, patients acquire resistance over time. The detailed mechanism(s) of this failure remain unclear and identification of new therapies for these patients is a serious challenge to physicians.

The majority of RCC is of the clear cell type RCC (ccRCC) that has a gender bias in incidence with male to female ratio of 2.3:1, suggesting that AR signals might play promotional roles for the RCC development and progression [5]. He et al. found that AR could promote RCC development and tumor metastasis via studies using various in vitro cell lines and in vivo mice models [6]. Interestingly, a recent survey of TCGA data with RCC suggested that AR might play a protective role in RCC development [7]. These contrasting results suggested AR might play distinct roles under different conditions during RCC progression.

Since hypoxia is a frequent occurrence in solid tumor masses that has functional consequences for tumor progression, we were interested in examining the potential AR roles under hypoxia during RCC progression. This is particularly relevant since a majority of ccRCC carry a mutation in the Von Hippel-Lindau (VHL), an E3 ubiquitin ligase that targets hypoxia inducible factor (HIF), thus implicating oxygen sensing as a critical factor for RCC development and progression [8].

Recently, it was suggested that hypoxia increased the size of the cancer stem-cells (CSCs) subpopulations and promoted the acquisition of a CSC-like phenotype [9]. It has also been suggested that CSCs contribute to tumor initiation, propagation, metastasis, and therapy failure [10]. Furthermore, CSCs have been shown to be resistant to conventional therapies, such as chemotherapy [11]. The stimulatory effects of hypoxia on tumorigenesis prompted us to investigate the underlying mechanisms by which hypoxia regulates the tumorigenic properties of CSC in RCC.

Here, we demonstrate that hypoxia decrease AR, which plays a critical role in regulating CSC formation through the long noncoding RNA lncTCFL5-2 which in turn regulates the SOX2 level through YBX1. Thus we delineate a novel pathway for hypoxia’s effects on CSC formation as well as provide a mechanistic explanation for the seemingly contrasting roles of AR in RCC development and progression.

## Materials and methods

### Cell culture

ACHN, OSRC-2, 293 T and SW839 cell lines were purchased from the American Type Culture Collection (ATCC, Manassas, VA, USA). All cell lines were cultured in Dulbecco's Modified Eagle's Media (Invitrogen, Grand Island, NY, USA) supplemented with 10% FBS (v/v), penicillin (25 units/ml), streptomycin (25 g/ml), 1% L-glutamine, and 10% fetal bovine serum (FBS). All cell lines were cultured in a 5% (v/v) CO_2_ humidified incubator at 37 ℃.

### Hypoxia

Hypoxia (0.5% O_2_, 5% CO_2_, 94.5% N_2_) was achieved using an In Vivo2 hypoxic workstation (Ruskinn Technologies) or in a positive pressure chamber receiving gas from a custom-mixed tank (Airgas).

### Human RCC specimens

30 patients from the Department of Shanghai Tenth People’s Hospital were enrolled in the study. None of the patients had received any preoperative treatment. Tumor size, stage (American Joint Committee on Cancer (AJCC), 2002 version) and grade (Furhman grade I to IV), were assembled in a computerized database for each participant. Three different types of tissues from each RCC patient were processed immediately after surgical resection: tumor-free tissue > 5 cm far from the tumor edge (N), adjacent nonmalignant tissues within 2 cm (AT), and tissues from the tumor (T). Areas of tissue necrosis and hemorrhage were excluded. The specimens from each patient were divided into two parts. One part was snap-frozen immediately after resection and stored in liquid nitrogen until they were used for experiments. The other part was preserved in 10% formaldehyde solution and then paraffin-embedded. This study was performed under a protocol approved by the Ethics Committee of Shanghai Tenth People’s Hospital, Tongji University School of Medicine (China). Written informed consent for participation in the study was obtained from each patient.

### Reagents and materials

GAPDH (6c5), AR (N-20) and YBX1 antibodies were purchased from Santa Cruz Biotechnology. CD24, CD133, and SOX2 antibodies were purchased from One World Lab. Anti-mouse/rabbit second antibody for Western Blot was from Invitrogen. Normal rabbit IgG was also from Santa Cruz Biotechnology.

### Lentivirus packaging

The pLVTHM-sh-lncTCFL5-2, pLVTHM-lncTCFL5-2, pPWI-YBX1, pLKO-sh-YBX1, pWPI-AR, pLVTHM-shSox2 or pLKO.1-shAR, the psAX2 packaging plasmid, and pMD2G envelope plasmid, were transfected into 293 T cells using the standard calcium chloride transfection method for 48 h to get the lentivirus soup. The lentivirus soups were collected and concentrated by density gradient centrifugation, then frozen in −80 ℃ for used immediately. The primers are presented in Additional file 1: Table S1.

### RNA extraction and qRT-PCR analysis

For RNA extraction, total RNAs were isolated using Trizol reagent (Invitrogen, Grand Island, NY). 1 µg of total RNA was subjected to reverse transcription using Superscript III transcriptase (Invitrogen, Grand Island, NY). Quantitative real-time PCR (qRT-PCR) was conducted using a Bio-Rad CFX96 system with SYBR green to determine the mRNA expression level of a gene of interest. Expression levels were normalized to the expression of GAPDH RNA. Quantitative real-time PCR (qRT-PCR) was conducted using a Bio-Rad CFX96 system with SYBR green to determine the mRNA expression level of a gene of interest. Expression levels were normalized to the expression of β-actin and/or GAPDH.

### Western blot analysis

Cells were lysed in RIPA buffer and proteins (30 µg) were separated on 8–10% SDS/PAGE gel and then transferred onto PVDF membranes (Millipore, Billerica, MA). After blocking membranes, they were incubated with appropriate dilutions of specific primary antibodies, the blots were incubated with HRP-conjugated secondary antibodies and visualized using ECL system (Thermo Fisher Scientific, Rochester, NY).

### Immunohistochemistry (IHC)

Human RCC sections at 5 μm and mice samples were deparaffinized in xylene solution and rehydrated using gradient ethanol concentrations. Endogenous peroxidase activity was blocked with 3% hydrogen peroxide in methanol for 10 min. Heat-induced antigen retrieval was performed for all sections with 0.01 M sodium citrate, pH 6.0, at 98 °C for 30 min. And IHC staining with specific primary antibodies against AR, YBX1, and SOX2 was performed.

### Immunofluorescence (IFC)

Different stable SW839 cell clones were cultured and fixed on 12 × 12 mm glass slides. After first incubating with antibodies specific for CD133 (One World Lab), and then incubating with antibodies specific for CD24 (One World Lab) then incubating with goat anti-rat IgG (Alexa Fluor 594, Invitrogen), and goat anti-mouse IgG (Alexa Fluor 488, Invitrogen), the slides were mounted by adding DAPI-Fluoromount-G (Southern Biotech, SBA, Birmingham, AL) and examined with a Zeiss axiophot photomicroscope (Carl Zeiss, Oberkochen, Germany).

### Sphere formation assay

The sphere formation assay was performed as described earlier. Briefly, single-cell suspensions (1 × 103, in 70 µl media) were mixed with 70 µl Matrigel (BD) and placed along the rim of the 24-well plates with three triplicate experiments. The culture plates were placed in 37 ℃ incubator for 10 min to let the mixture solidify and 500 µl media was then added into the wells. Sphere numbers were counted after 7 ~ 14 days under Olympus light microscope and size differences were also examined.

### Chromatin immunoprecipitation Assay (ChIP)

Cells were cross-linked with 4% formaldehyde for 10 min followed by cell collection and sonication with a predetermined power to yield genomic DNA fragments of 300–1000 bp long. Lysates were precleared sequentially with normal rabbit IgG (sc-2027, Santa Cruz Biotechnology) and protein A agarose. Anti-AR antibody and Anti-YBX1 (2.0 µg) was added to the cell lysates and incubated at 4 °C overnight. For the negative control, IgG was used in the reaction. Specific primer sets designed to amplify a target sequence within human lncTCFL5-2 and SOX2 promoter; PCR products were identified by agarose gel electrophoresis (for detailed information of the locations of each ARE see the Additional file 1: Table S1).

### RNA immunoprecipitation (RIP)

Native RIP was performed as described previously. Briefly, SW839 or OSRC-2 cells were lysed in RIPA lysis buffer (20 mM Tris–HCl pH 7.5, 150 mM NaCl, 1 mM Na2 EDTA, 1 mM EGTA, 1% NP-40, 1% sodium deoxycholate, 2.5 mM sodium pyrophosphate, 1 mM beta-glycerophosphate, 1 mM Na_3_VO_4_ and 1 µg/ml leupeptin) supplemented with anti-RNase, protease inhibitor cocktail. RNase-free DNase (NEB) (400U) was then added to the lysates and incubated on-ice for 30 min. The cell lysates were diluted in the RIPA buffer and 50 µl of the supernatant was saved as input for PCR analysis. 500 µl of the supernatant was incubated with 4 µg of AR and YBX1 antibody (normal rabit IgG overnight as control). Protein A/G beads were pre-blocked by 15 mg/ml BSA in PBS. Then pre-blocked beads were added to the antibody-lysate mixture and incubated for another 2 h. The RNA/antibody complex was washed four times by RIPA buffer supplemented with anti-RNase, protease inhibitor cocktail. The RNA was extracted using Trizol (Invitrogen) according to the manufacturer’s protocol and subjected to RT-qPCR analysis. For UV cross-linking and RIP, cells were first subjected to UV cross-linking (200 mJ/cm2) and then conducted as native RIP protocol.

### Luciferase assay

The human promoter region of lncTCFL5-2 and SOX2 were constructed into pGL3-basic vector (Promega, Madison, WI, USA). Cells were plated in 24-well plates and the cDNAs were transfected using Lipofectamine (Invitrogen) according to the manufacturer’s instructions. pRL-TK was used as internal control. Luciferase activity was measured by Dual-Luciferase Assay (Promega) according to the manufacturer’s manual.

### MTT assay

Cell proliferation rates were measured using MTT. After 24 h transfection with plasmids, SW839 or OSRC-2 cells were seeded at 1000/well in 96-well plates. The cell proliferation assay was performed on days 1, 2, 3 and 4. MTT reagent was added to each well, then the plates were incubated for 2 h at 37 ℃. Before the endpoint of incubation, the absorbance was measured at 450 nm. Each sample was assayed in triplicate.

### Animal study

OSRC-2 cells expressing pLVTHM (2X10^6^) and OSRC-2 cells expressing sh-lncTCFL5-2 (2X10^6^) were divided into two groups. One group was cultured under hypoxia for 48 h and the other group cultured under normoxia for 48 h. Then the cells were injected into left renal capsule of 6-week-old male athymic nude mice (NCI) (n = 8 mice per group). Once the tumor formation was detectable, we treated the hypoxia groups with and without lncTCFL5-2 Sunitinib 40 mg/kg every day for two weeks, After 6–8 weeks, mice were sacrificed, and tumors were excised and weighed. Studies on animals were conducted with approval from the Animal Research Ethics Committee of the University of Rochester Medical Center.

### TCGA data analysis

The RNA-seq data about cohorts of 604 clear cell RCC (ccRCC), cases were extracted from TCGA (https://xena.ucsc.edu/) as well as their clinical outcome. The RCC patients were divided into two groups based on the levels of AR mRNA expression. Kaplan–Meier analysis was performed using Xena (http://xena.ucsc.edu/). The AR expression in different stages and differents grade were also analyzed using GraphPad Prism.

### IVIS

Male 6 weeks old Balb/c nude mice divided into four groups (eight mice per group) for injection with Luc tranduced OSRC-2 cells, which were pre-cultured as described in the animal study. Then 1 × 10^6^ of the prepared stable clones o OSRC-2-Luc cells suspended in 100 μL PBS were injected into the caudal artery of anesthetized mice using 29 G syringe needle in a short time (< 3 s) 0.28 Noninvasive In Vivo Fluorescent Imager (IVIS Spectrum, Caliper Life Sciences, USA) and X-ray (Faxitron, Tucson, USA) were used to capture the images once a week. After 4 weeks, tumors as well as metastatic lesions were removed and counted for subsequent analysis.

### Statistics

All statistical analyses were carried out with SPSS 19.0 (SPSS Inc, Chicago, IL). The data values were presented as the mean ± SD. Differences in mean values between two groups were analyzed by two-tailed Student’s t test and the mean values of more than two groups were compared with one way ANOVA. p ≤ 0.05 was considered statistically significant.

## Results

### Hypoxia suppresses AR expression and increases the CSC formation in RCC

**Fig. 1:**
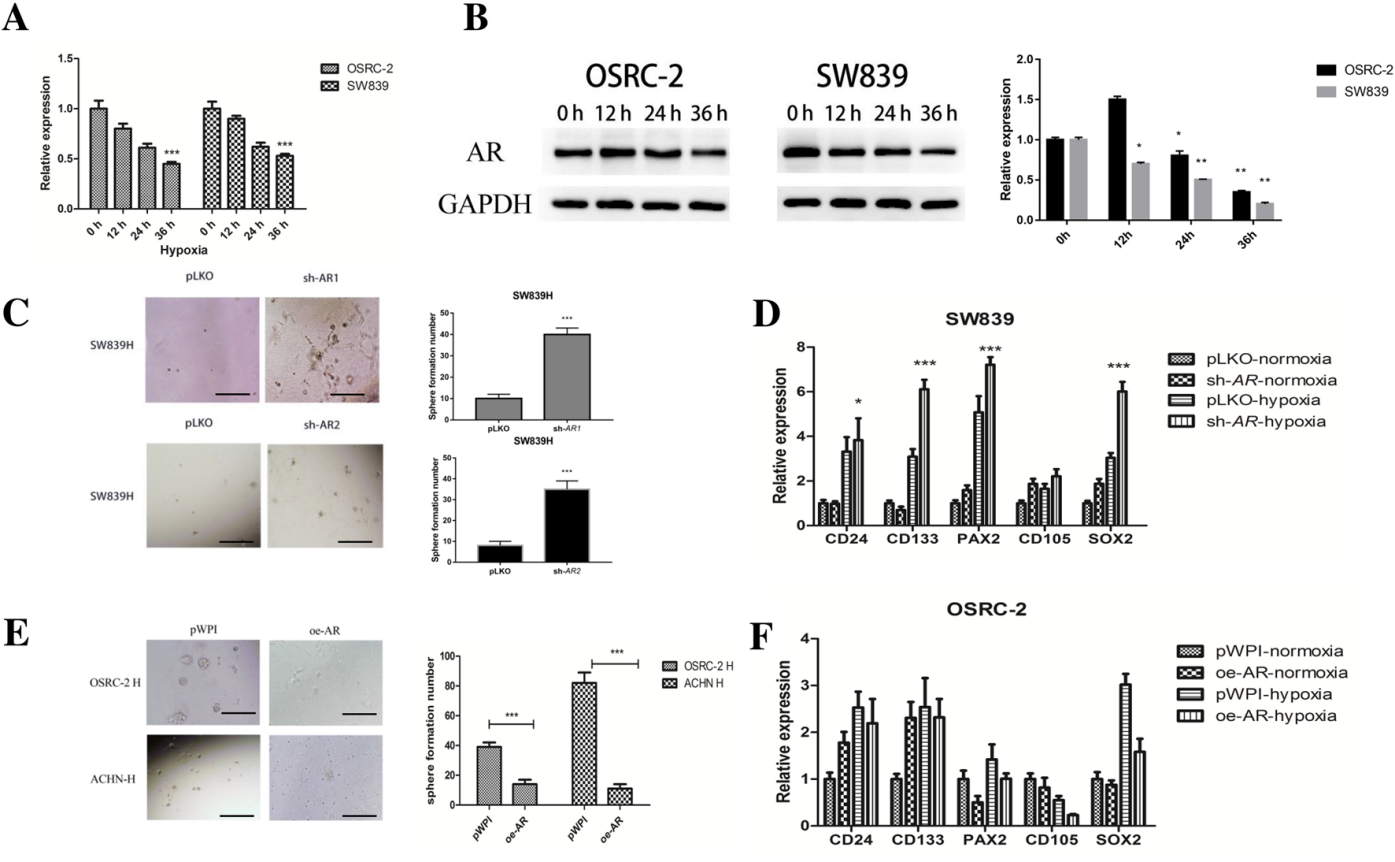
Hypoxia (1% oxygen) suppresses AR expression and increases the CSC formation in RCC cells. **A** SW839 and OSRC-2 cells were exposed to hypoxia for 0 h, 12 h, 24 h, and 36 h. Real-time PCR was used to detect AR expression. **B** SW839 and OSRC-2 cells were exposed to hypoxia for 0 h, 12 h, 24 h, and 36 h. Western-blots were used to detect AR expression. **C** SW839 and SW839 with sh-AR1 and sh-AR2 were exposed to hypoxia (H) for 2 days, sphere formation assays were performed to evaluate the CSCs number. After 14 days incubation, colonies in five random fields per each well were counted under a microscope. **D** SW839 cells were virally transduced with sh-AR and cells exposed to hypoxia (H) or normoxia (N) for 2 days. Total RNAs were analyzed for CSCs markers including *CD24, CD133, PAX2, CD105, SOX2* by real-time PCR. **E** OSRC-2 and ACHN cells were virally transduced with overexpressed AR and exposed to hypoxia (H) for two days, sphere formation assays were performed to evaluate the CSCs number. After 14 days incubation, colonies in five random fields per each well were counted under a microscope. **F** OSRC-2 cells were virally transduced with oe-AR and cells were exposed to hypoxia (H) or normoxia (N) for 2 days. Total RNAs were analyzed for CSCs markers including CD24, CD133, PAX2, CD105 and SOX2 by real-time PCR.(AR: androgen receptor; GAPDH: Recombinant protein of human glyceraldehyde-3-phosphate dehydrogenase; pLKO: pLKO.1-puro plasmid; sh-AR1/2: The plasmid encoding AR-shRNA1/2; pWPI: Addgene plasmid 12,254,; oe-AR: The plasmid encoding AR sequence, H:hypoxia; N: normoxia. * P<0.05, ** P<0.01, ***P<0.001)

We first examined hypoxia’s influence on the AR expression, and found hypoxia could decrease AR mRNA after 24 h in both RCC OSRC-2 and SW839 cells (Fig. 1A). Western-blot analysis also confirmed hypoxia could decrease AR protein expression after 36 h in both RCC OSRC-2 and SW839 cells (Fig. 1B and Additional file 1: Fig. S1A). Because hypoxia can suppress gene translation in some cases, we tested additional genes (EZH2 and SRC) to determine their protein expression level. The results showed that EZH2 decreased while SRC increased under hypoxia condition (Additional file 1: Fig. S1B). These results demonstrated that hypoxia-decreased AR may not be the result of general translation suppression.

We then examined the consequence of hypoxia-suppressed AR signaling on the RCC progression, with a focus on CSC phenotype, as abundant evidence indicated that CSC might play important roles for tumor development and progression [12].

Using sphere formation assays to evaluate the CSC formation, we found knocking down AR (*sh-AR*) using two different sequences both increased the CSC formation (Fig. 1C) as well as the expression of CSCs biomarkers including *CD24, CD133, PAX2, SOX2* and *CD105* in RCC SW839 cells under hypoxia condition (Fig. 1D). In the following experiments we just used one sh-AR sequence. In contrast, overexpressing AR decreased the CSC formation (Fig. 1E) and expression of CSC biomarkers, including *CD24, CD133, PAX2, SOX2* and *CD105* in OSRC-2 cells under hypoxia (Fig. 1F). The efficiency of sh-AR and overexpression AR (oe-AR) were tested and shown in Additional file 1: Fig. S1C. We also confirmed the hypoxia condition by testing the protein of HIF-1α expression, the results demonstrated that hypoxia can decrease HIF-1α protein expression dramatically in OSRC-2 cells (Additional file 1: Fig. S1D).

Together, results from Fig. 1A–F and Additional file 1: Fig. S1A–C suggest that through suppression of AR expression, hypoxia may increase the CSC phenotype in RCC cells.

### Hypoxia-suppressed AR expression may alter the expression of lncRNAs in RCC cells

**Fig. 2:**
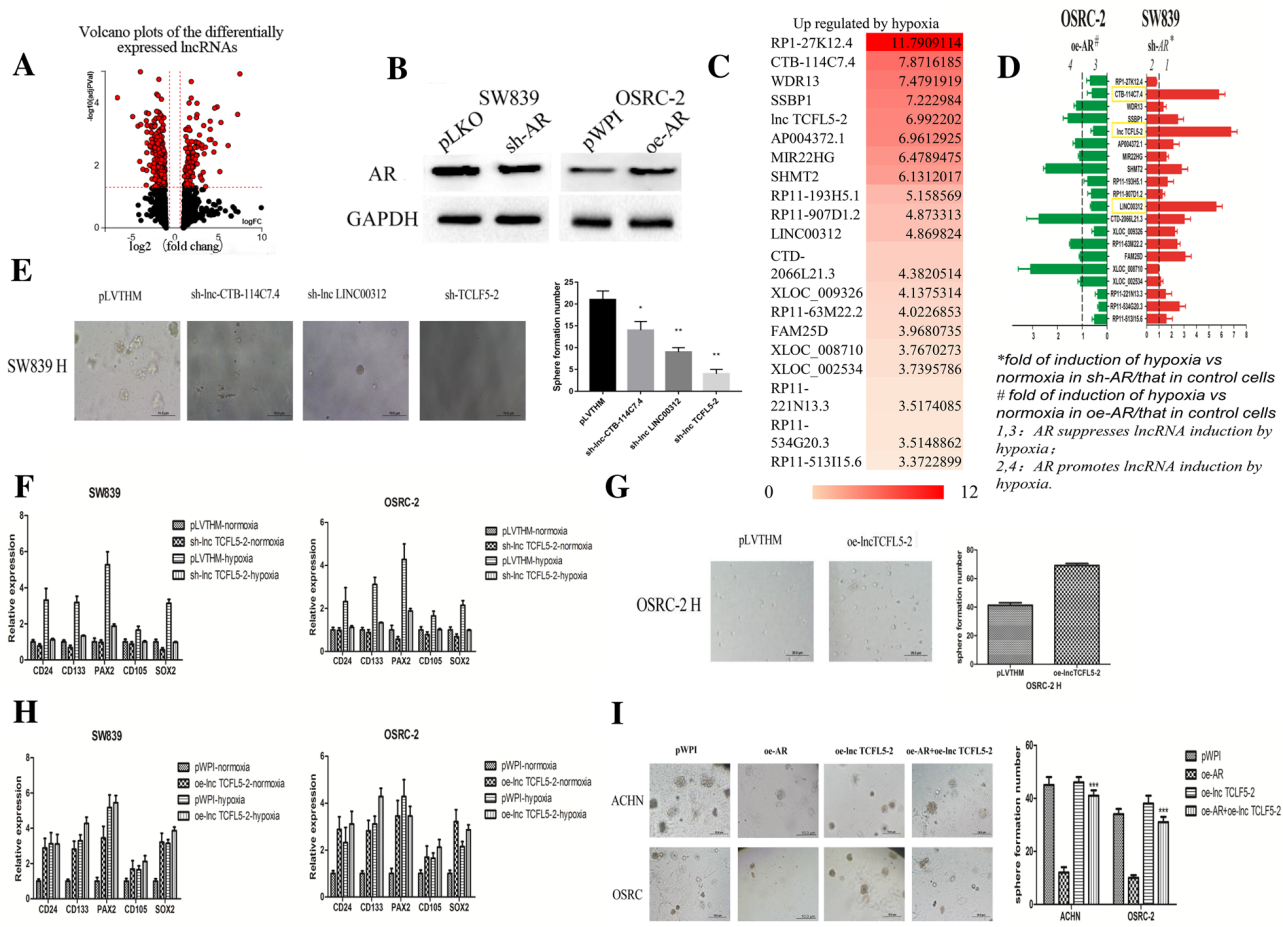
Hypoxia-suppressed AR expression may alter the expression of lncRNAs in RCC cells. **A** SW839 cells, pre-cultured in the hypoxia condition (1% oxygen level for 24 h) and normoxia condition. Volcano plots of the differentially expressed lncRNAs: red spots represent fold change more than 0.6 and P valve less than 0.05. **B** Western-blot showed the efficiency of sh-AR in SW839 cells and overexpressed AR (oe-AR) in OSRC-2 cells. **C** The list of top 20 upregulated lncRNAs by hypoxia. **D** SW839 and OSRC-2 cells were virally transduced with sh-AR or oe-AR, and then cells were exposed to hypoxia (H) or normoxia (N) for 2 days. Total RNAs were analyzed by Q-PCR to show these 20 lncRNAs expression. The results showed that three lncRNAs (lnc-CTB-114C7.4, lnc-TCFL5-2(XLOC_013838)and LINC00312) were suppressed by AR. **E** SW839 cells were virally transduced with pLVTHM, sh-lnc-CTB-114C7.4, sh-lncTCFL5-2, sh-LINC00312 and cells were exposed to hypoxia (H) for 2 days. Sphere formation assay were performed to evaluate the CSCs numbers. After 14 days of incubation, colonies in five random fields per each well were counted under a microscope. **F** SW839 and OSRC-2 cells were virally transduced with sh-lncTCFL5-2 and then cells were exposed to hypoxia and normoxia for 2 days. Total RNAs were analyzed for CSCs markers CD24, CD133, PAX2, CD105, SOX2 by real-time PCR. **G** OSRC-2 cells were virally transduced with oe-lncTCFL5-2 and then cells exposed to hypoxia (H) for 2 days. Sphere formation assay were performed to evaluate the CSCs numbers. **H** SW839 and OSRC-2 cells were virally transduced with oe-lncTCFL5-2 and then cells exposed to hypoxia and normoxia for 2 days. Total RNA were analyzed for CSCs markers CD24, CD133, PAX2, CD105, SOX2 by real-time PCR. **I** ACHN and OSRC-2 cell were virally transduced with oe-AR or were co-transfected with oe-AR and oe-lncTCFL5-2, and then cells exposed to hypoxia for 2 days. Sphere formation assays were performed to evaluate the CSCs phenotype. (AR: androgen receptor; pLVTHM: Addgene plasmid 12,247; sh-lnc-CTB-114C7.4: The pLVTHM plasmid encoding lnc-CTB-114C7.4 shRNA, sh-lncTCFL5-2: The pLVTHM plasmid encoding lncTCFL5-2 shRNA, sh-LINC00312: The pLVTHM plasmid encoding LINC00312 shRNA; oe-AR: The plasmid encoding AR sequence; oe-lncTCFL5-2: The plasmid encoding lncTCFL5-2 sequence.)

To dissect the molecular mechanisms how hypoxia-suppressed AR can alter the RCC CSCs phenotype, we focused on the long noncoding RNAs (lncRNAs) as recent studies indicated that they might play important roles to promote tumor progression in a variety of settings. Using lncRNA microarray analysis, we subjected SW839 cells, a VHL-mutant RCC cell line, to hypoxia (1% oxygen level for 24 h) and carried out a comparative analysis of the expression of 40,000 lncRNAs and found the expression of nearly 7000 lncRNAs was increased, with a reduction of expression of nearly 13,000 lncRNAs in response to hypoxia with a subset of lncRNAs that showed more than two fold difference in expression (Fig. 2A and Additional file 1: Fig. S2A). We then focused on those lncRNAs with significant changes of expression in response to hypoxia (fold change > 0.6, and P value < 0.05), and chose 20 up-regulated lncRNAs and 20 down-regulated lncRNAs for further study (Fig. 2C and Additional file 1: Fig. S2B, C).

Next, to link those 40 hypoxia-altered lncRNAs to the AR-modulated CSC phenotype under hypoxia, we altered AR expression (Fig. 2B) and examined its impact on these 40 lncRNAs, and results revealed that knocking down AR in SW839 cells (Fig. 2D) significantly altered the expression of some lncRNAs including lnc-CTB-114C7.4, lncTCFL5-2(XLOC_013838) and LINC00312 suggesting AR has an effect on the hypoxia-induced lncRNAs expression. Consistent with this finding, overexpression of AR in OSRC-2 cells suppressed the hypoxia-induction of these 3 lncRNAs (Additional file 1: Fig. S2D). Similarly, we found that AR regulates hypoxia-induced suppression of certain lncRNAs (Additional file 1: Fig. S2C).

Together, results from Fig. 2A-D and Additional file 1: Fig. S2A–D suggest that hypoxia can decrease AR expression, which may significantly alter expression of some special lncRNAs.

### Hypoxia-decreased AR may result in de-repression of lncTCFL5-2, which contributes the CSC formation in RCC cells

To examine the role of AR-regulated lncRNAs in hypoxia-induced CSC phenotypes in RCC, we altered the expression of these 3 lncRNAs and found that knocking down lncTCFL5-2 with two different sh-sequence plasmids could both significantly suppress RCC sphere formation under the influence of hypoxia (Fig. 2E, Additional file 1: Fig. S2E). So we did the following experiments just using one sh-sequence plasmid.

Furthermore, knocking down lncTCFL5-2 also decreased expression of CSC related genes, including *CD24, CD133, PAX2, SOX2* and *CD105* in SW839 and OSRC-2 cells under hypoxia (Fig. 2F). In contrast, overexpressing lncTCFL5-2 increased CSC sphere formation (Fig. 2G) as well as expression of CSCs biomarkers, including *CD24, CD133, PAX2, SOX2* and *CD105* in SW839 and OSRC-2 under normoxia but not in a hypoxia condition (Fig. 2H).

Using interruption approaches, we also found exogenous overexpression of lncTCFL5-2 reversed/blocked AR-decreased CSC formation in both RCC OSRC-2 and ACHN cells under hypoxia (Fig. 2I). To further confirm AR effect on the CSC population through lncTCFL5-2, we also tested the relationship between AR and lncTCFL5-2 in Additional file 1: Fig. S2F, and found that the lncTCFL5-2 cannot change AR at either mRNA level or protein level.

Together, results from Fig. 2E–I and Additional file 1: Fig. S2E-F suggest that hypoxia-suppressed AR may result in de-repression of lncTCFL5-2, which may then increase the CSC population in RCC cells.

### Mechanism dissection how AR suppresses lncTCFL5-2 expression

**Fig. 3:**
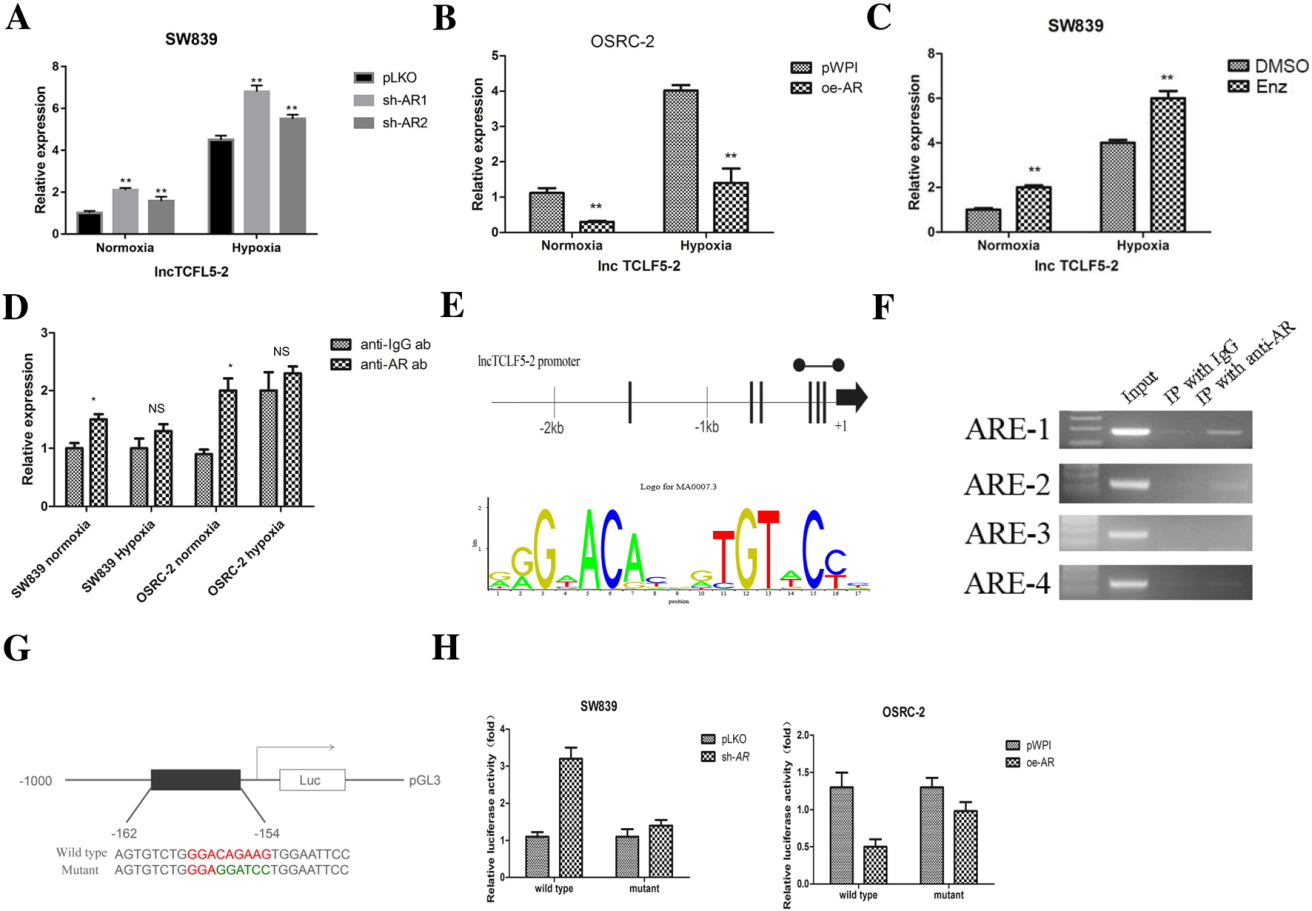
Mechanism dissection how AR suppresses lncTCFL5-2 expression. **A** SW839 cell were virally transduced with sh-AR1, sh-AR2 and cells were exposed to hypoxia or normoxia for 2 days. qPCR analysis of the expression of lncTCFL5-2. **B** OSRC-2 cells were virally transduced with oe-AR and cells exposed to hypoxia or normoxia for 2 days, qPCR analysis of the expression of lncTCFL5-2. **C** SW839 cells were treated with Enzalutamide (Enz) 10 µM and then cells exposed to hypoxia or normoxia for 2 days, qPCR analysis of the expression of lncTCFL5-2. **D** The interaction between AR and lncTCFL5-2 in both SW839 and OSRC-2 cells under normoxia and hypoxia condition were detected by RIP. **E** Bioinformatic analysis of potential AR binding sites in lncTCFL5-2 promoter. **F** Lysates of OSRC-2 cells were subjected to ChIP assay. ChIP products were amplified by PCR reaction. **G**, **H** SW838 and OSRC-2 cells were co-transfected with the indicated reporter constructs and Renilla luciferase plasmid. 48 h after transfection, reporter activity was then measured and plotted after normalizing against the Renilla luciferase activity (mean ± SD).. (pLKO: pLKO.1-puro plasmid, sh-AR1/2: The plasmid encoding AR-shRNA1/2, oe-AR: The plasmid encoding AR sequence, pWPI: Addgene plasmid 12,254, ARE:AR Element)

Based on the lncipedia data (https://lncipedia.org/db/gene/lnc-TCFL5-2), lncTCFL5-2 is encoded by the gene located in chromosome 20 (hg38) from 62,782,754–62,794,246 with a length of 463 bases. To dissect mechanisms how AR regulates lncTCFL5-2 expression, we first found knocking down AR increased the lncTCFL5-2 expression in SW839 cells (Fig. 3A), and overexpressing AR decreased the lncTCFL5-2 expression in OSRC-2 cells (Fig. 3B). Similar results were obtained when we replaced OSRC-2 cells with ACHN cells (Additional file 1: Fig. S3A). Importantly, treating with the anti-androgen enzalutamide to replace AR-shRNA, we also obtained similar results showing AR inactivation increased lncTCFL-5–2 expression (Fig. 3C). Consistent with this, there was increased expression of CSCs marker genes including *CD24, CD133, PAX2, SOX2* and *CD105* in OSRC-2 cells treated with Enzalutamide (Additional file 1: Fig. S3B).

To further examine whether AR can regulate lncTCFL5-2 expression through transcriptional or post-transcriptional regulatory mechanisms, we tested whether AR could bind to the lncRNA. The RIP assay with anti-AR antibody together with IgG control followed by analysis of the associated RNA through reverse-transcription and quantitative PCR indicated that there was slight increase of AR association with the lncRNA compared to the negative control in both normoxia and hypoxia conditions (Fig. 3D). Thus, we concluded that AR unlikely interacted with the lncRNA in a functionally significant manner. On the other hand, through bioinformatics analysis (Ensembl and PROMO 3.0) of AR binding sites in the potential lncRNA promoter, we found 4 potential AREs within 2.5 kb upstream of the transcriptional start site (Fig. 3E). Chromatin immunoprecipitation (ChIP) assay with anti-AR antibody in OSRC-2 cells revealed that AR could bind to the ARE1 located on the 162nt to 154nt upstream of the transcription start site of lncTCFL5-2 in OSRC-2 cells (Fig. 3F).

Importantly, using luciferase reporter construct with 1 kb upstream sequence containing normal (wild type) or mutated ARE-1, we found overexpressing AR significantly decreased the reporter activity in OSRC-2 cells and sh-AR significantly increased the reporter activity in SW839 cells transfected with the wild type construct but not the mutant construct (Fig. 3G, H).

Together, results from Fig. 3A–H and Additional file 1: Fig. S3A-B demonstrated that AR could suppress lncTCFL5-2 expression at a transcriptional level via binding to the ARE-1 located in its 5’ promoter region.

### Mechanism dissection how AR-suppressed lncTCFL5-2 may increase CSC phenotype via interacting with the YBX1 in RCC

**Fig. 4:**
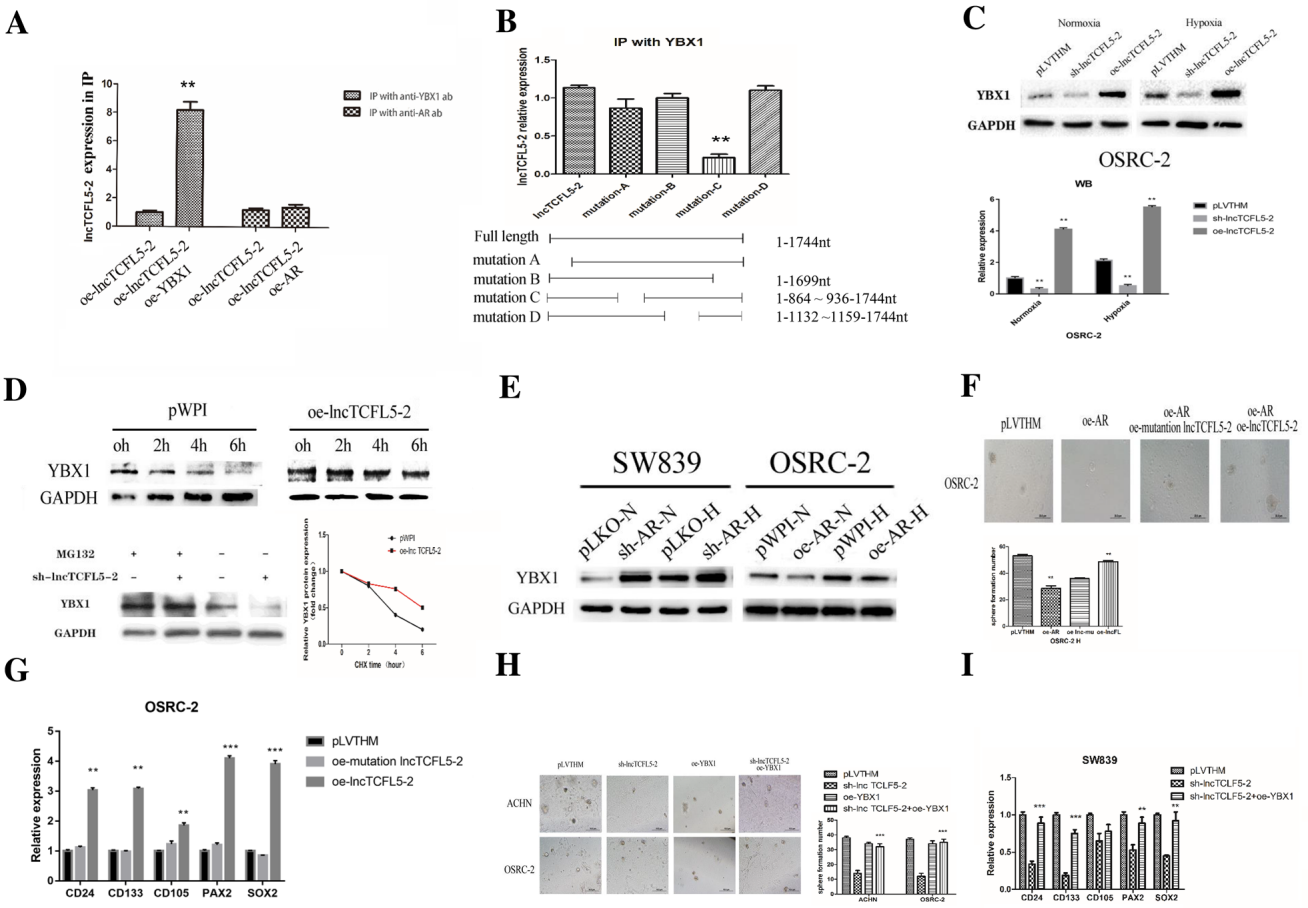
Mechanism dissection how AR-suppressed lncTCFL5-2 may increase CSCs the phenotype via interacting with the YBX1 in RCC. **A** 293 T cells were co-transfected with oe-AR and oe-lncTCFL5-2 or oe-YBX1 and oe-lncTCFL5-2, RIP detected the interaction between YBX1 and lncTCFL5-2. **B** 293 T cells were co-transfected with oe-YBX1 and four deletions mutants of lncTCFL5-2, RIP detected the interaction between YBX1 and the four mutants. **C** OSRC-2 cells were lentivirally transduced with sh-lncTCFL5-2 or oe-lncTCFL5-2, and then exposed to hypoxia or normoxia for 2 days. Western blot analysis of the expression of YBX1. **D** OSRC-2 cells were lentivirally transduced with pWPI or oe-lncTCFL5-2, then treated with 10 mg/ml cycloheximide (CHX). YBX1 protein levels were analyzed by western blot (lower right panel). The lower left panel is a graphic representation of YBX1 metabolic stability. MG132 (20 µM) was added in the OSRC-2 cells, Western blot analysis of the expression of YBX1. **E** SW839 and OSRC-2 cells were lentivirally transduced with sh-AR or oe-AR, respectively, and then cells were exposed to hypoxia or normoxia for 2 days. Western blot analysis of the expression of YBX1. **F** OSRC-2 cells were lentivirally transduced with oe-AR or oe-AR together with wildtype or mutant lncTCFL5-2. Sphere formation assays were performed to evaluate the CSC phenotype. After 14 days of incubation, colonies in five random fields per well were counted under a microscope. **G** OSRC-2 cells were lentivirally transduced with pLVTHM or wildtype or mutant lncTCFL5-2. Total RNAs were analyzed for CSC markers CD24, CD133, PAX2, CD105, and SOX2 by real-time PCR. **H** ACHN and OSRC-2 cells were lentivirally transduced with sh-lncTCFL5-2 or were co-transduced with sh-lncTCFL5-2 and oe-YBX1, and then cells exposed to hypoxia for 2 days. Sphere formation assay were performed to evaluate the CSC phenotype. After 14 days of incubation, colonies in five random fields per well were counted under a microscope. **I** SW839 cells were lentivirally transduced with sh-lncTCFL5-2 or co-transfected with sh-lncTCFL5-2 and oe-YBX1 and then cell exposed to hypoxia and normoxia for 2 days. Total RNAs were analyzed for CSC marker CD24, CD133, PAX2, CD105, SOX2 by real-time PCR (ab: Antibody, pLVTHM: Addgene plasmid 12,247; sh-lncTCFL5-2: The pLVTHM plasmid encoding lncTCFL5-2 shRNA, oe-lncTCFL5-2: The plasmid encoding lncTCFL5-2 sequence, oe-YBX1: The plasmid encoding YBX1 sequence; N: normoxia, H: hypoxia)

The lncRNAs have been implicated in a variety of cellular functions broadly through specific mechanisms, such as signaling molecules, decoys, guides, and scaffolds to support multi-protein structures [13]. Among many possibilities suggesting that lncRNA-TCFL-2 can regulate CSC formation, we tested whether this lncRNA can interact with a protein known to be involved in CSC formation. Through a bioinformatic analysis of likely protein-RNA interactions (RBP map), we found that lncTCFL5-2 might be able to interact with YBX1, a protein reported to be a regulator of SOX2, a transcriptional factor critical for maintaining stem cell characteristics [14]. Results from RIP assays revealed that lncTCFL5-2 indeed interacted with YBX1 compared to AR that failed to interact with this lncRNA, consistent with the previous data (Fig. 4A, Fig. 3D). Furthermore the lncTCFL5-2 and YBX1 interaction could be demonstrated with the endogenous level in OSRC-2 cells, not through overexpression in either of them (Additional file 1: Fig. S3C, D).

To further solidify the functional significance of lncTCFL5-2 with YBX1, we also mapped the YBX1 binding sequence in the lncRNA. There are 4 putative YBX1-binding areas with predicted binding motifs in the lncTCFL5-2. Through deletion analysis, we found that the 864-936nt region with 6 binding motifs (mutation-C) is critical for lncRNA-YBX1 interaction (Fig. 4B) while the remaining single binding motif in the 5’ and 3’ ends, as well as one single binding motif at 1150nt, appear not important for binding to YBX1.

Importantly, we found that YBX1 protein expression was also influenced by hypoxia and lncTCFL5-2 high expression (Fig. 4C). To directly test whether lncTCFL5-2 could regulate YBX1 protein stability, cycloheximide (CHX) was used to block the de novo protein synthesis, and the expression of YBX1 protein in OSRC-2 cells was tracked at various time points after CHX addition. We found the overexpression of lncTCFL5-2 prolonged YBX1 protein half-life (Fig. 4D). In addition, this increase of YBX1 stability is likely due to reduced proteasome-mediated degradation as inhibitor of proteasome, MG132, could increase the YBX1 level, more dramatically in cells with a knock down of lncTCFL5-2 (Fig. 4D lower left panel). It is likely that ubiquitin-mediated degradation plays a role in the protection by lncTCFL5-2 on YBX1 although the exact E3 ligase remains to be determined.

The AR/lncTCFL5-2 connection suggested that AR might regulate YBX1 expression and indeed we found overexpressing AR in ORSC-2 cells resulted in repressing the YBX1 expression in SW839 cells likely through repression of lncTCFL5-2 expression (Fig. 4E). Importantly, using an interruption approach, we found AR-suppressed CSCs formation could be reversed by overexpression of the wildtype lncTCFL5-2, but not by a mutant lncTCFL5-2 failed to bind to YBX1 in OSRC-2 cells (Fig. 4F), further solidifying the significance of lncTCFL5-2 and YBX1 interaction in regulating the CSCs phenotype. To confirm this interpreted result we tested the AR and lncTCFL5-2 levels by qPCR (Additional file 1: Fig. S3E).

The expression of CSC biomarkers in response to wildtype and mutant lncTCL5-2 also supported this conclusion (Fig. 4G). Furthermore, a reduction of lncTCFL5-2 resulted in reduced RCC sphere formation under hypoxia, and this reduction can be reversed by simultaneous exogenous expression of YBX1 (Fig. 4H). Similar results were also obtained when we replaced sphere formation assay with the measurement of the expression of CSC biomarkers (Fig.  4I).

Together, results from Fig.  4A–I and Additional file 1: Fig. S4C-E demonstrated that the AR/lncTCFL5-2/YBX1 signaling axis plays a critical role to regulate the CSC phenotype in RCC cells in response to hypoxia.

### Mechanism dissection how AR/lncTCFL5-2/YBX1 signals may function via modulating SOX2 to alter the CSCs formation in RCC

**Fig. 5:**
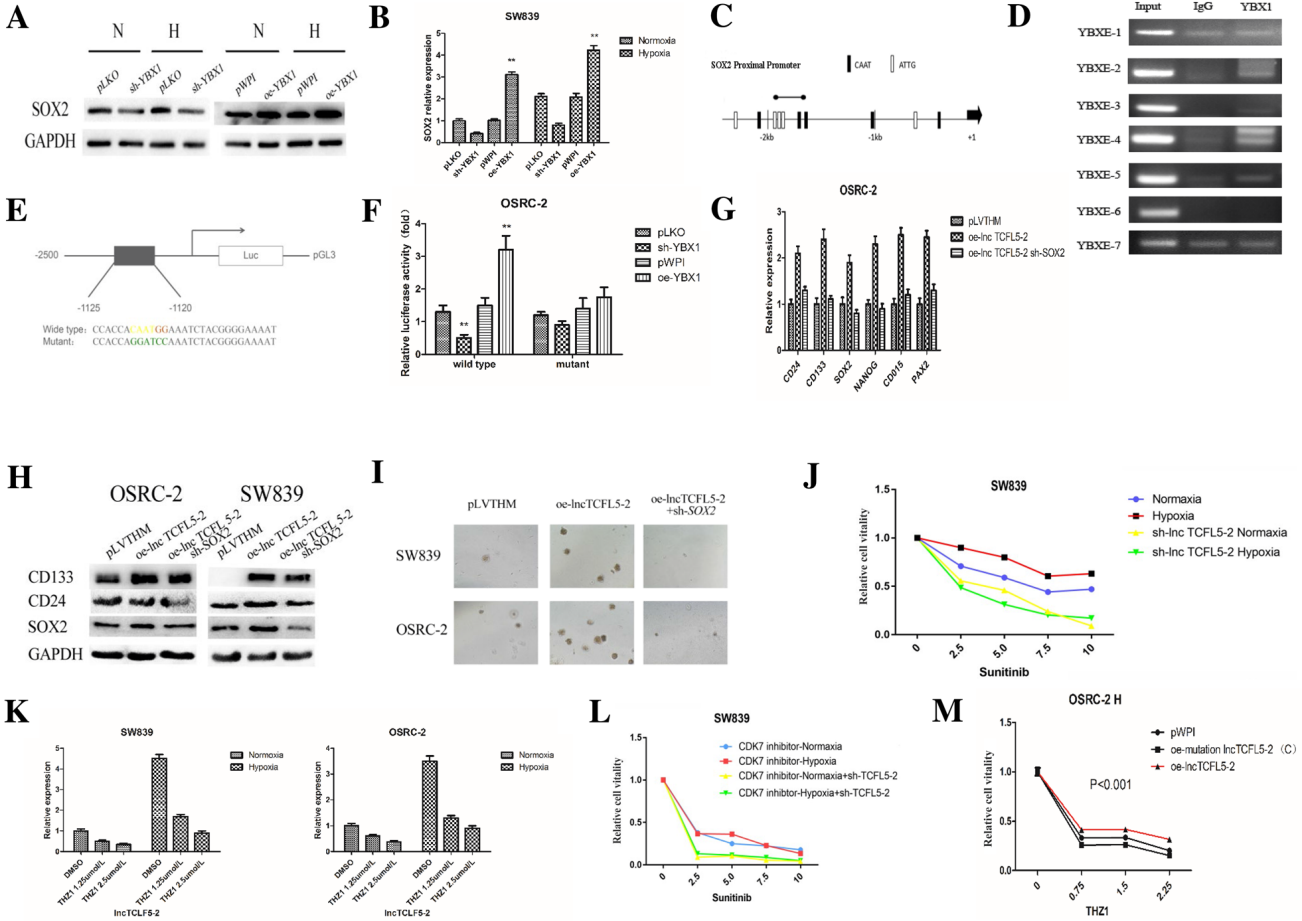
Mechanism dissection how AR/lncTCFL5-2/YBX1 signals may function via modulating SOX2 to alter the CSCs formation and chemotherapy resistance in RCC cells. **A**, **B** SW839 cells were virally transduced with sh-YBX1 or oe-YBX1 and then exposed to hypoxia and normoxia for 2 days. SOX2 expression was evaluated by western blot (**A)** and qPCR (**B**). **C** Bioinformatic analysis of potential YBX1 binding sites in SOX2 promoter. **D** Lysates of OSRC-2 cells were subjected to ChIP assay. ChIP products were amplified by PCR reactions. **E**, **F** Co-transfection of SOX2 promoter constructs containing the wild type or mutant region into OSRC-2 cells and luciferase assay was applied to detect the luciferase activity. **G** OSRC-2 cells were lentivirally transduced with pLVTHM or oe-lncTCFL5-2 or co-transfected with oe-lncTCFL5-2 and sh-SOX2. Total RNAs were analyzed for CSCs markers CD24, CD133, SOX2, NANOG, and CD105, PAX2, by real-time PCR. **H** SW839 and OSRC-2 cells were lentivirally transduced with pLVTHM or oe-lncTCFL5-2 or co-transfected with oe-lncTCFL5-2 and sh-SOX2. Western blot analysis of the expressions of CD24, CD133, SOX2. **I** SW839 and OSRC-2 cells were lentivirally transduced with pLVTHM or oe-lncTCFL5-2 or co-transfected with oe-lncTCFL5-2 and sh-SOX2. Sphere formation assays were performed to evaluate the CSC phenotype. After 14 days of incubation, colonies in five random fields per each well were counted under a microscope. **J** MTT assay was used to determine viability of cells exposed to hypoxia comparing to normoxia in SW839 cells with sh-lncTCFL5-2 or without sh-lncTCFL5-2 with Sunitinib treatment. **K** SW839 and OSRC-2 cells were exposed to hypoxia and normoxia for 2 days, and then treated with THZ1. Total RNAs were analyzed for lncTCFL5-2 by real-time PCR. **L** SW839 cells were lentivirally transduced with or without sh-lncTCFL5-2, then exposed to hypoxia and normoxia, treated with DMSO or Sunitinib combined with CDK-7 inhibitor. MTT assay was used to detect the cell viability. **M** OSRC-2 cells were virally transduced with pWPI or wildtype or mutant lncTCFL5-2, then treated with different concentrations of THZ1 followed by MTT assay

To further dissect the mechanism of SOX2 regulation by YBX1, we focused on the potential transcriptional regulation of SOX2 by YBX1 as the latter could regulate SOX2 expression at both mRNA and protein levels in SW839 cells (Fig. 5A, B). Consistent with a previous report [14], bioinformatics analysis indicated that there are 10 putative YBX1 response elements (YBX1REs) located within 2.5 kb *SOX2* promoter region (Fig. 5C). Chromatin immunoprecipitation (ChIP) assay with anti-YBX1 antibody revealed that YBX1 could bind to the YBX1RE located on the 1600–2000 nt region upstream of the transcription start site of SOX2 in OSRC-2 cells (Fig. 5D).

We therefore constructed a luciferase reporter construct bearing 2.5 kb SOX2 promoter sequence as well as a mutant reporter construct with mutations in the YBX1REs (see details in Fig. 5E). As expected, results from the luciferase reporter assay revealed that expression of YBX1 significantly increased reporter luciferase activity in OSRC-2 cells transfected with wild type SOX2 promoter, but not in the cells with mutant SOX2 promoter (Fig. 5F).

To further demonstrate the functional significance of SOX2 expression in RCC CSCs formation, we examined the consequence of SOX2 expression in regulating CSC biomarker expression. The results revealed that the hypoxia-induced CSC phenotype is strongly correlated with the appearance of positive expression of CD24 and CD133 (Additional file 1: Fig. S3F). Significantly, knocking down SOX2 resulted in repression of CSCs biomarkers including mRNA levels of CD24, CD133, SOX2, NANOG, CD105 and PAX2, in OSRC-2 cells  (Fig. 5G), protein levels of CD133, CD24 and SOX2 in both OSRC-2 and SW839 cells (Fig. 5H), as well as sphere formation under hypoxia of SW839 and OSRC-2 cells (Fig. 5I).

Together, results from Fig.  5A–I suggest that the AR/lncTCFL5-2/YBX1 signaling axis regulates RCC CSC phenotypes via modulating the SOX2 expression.

### AR/lncTCFL5-2/YBX1/SOX2-modulated CSC formation led to alter the chemotherapy resistance in RCC cells

To determine the biological consequence of the AR/lncTCFL5-2/YBX1/SOX2 signaling axis under hypoxia in the RCC progression, we focused on the chemotherapy resistance, since early studies indicated that CSCs formation is often negatively correlated with therapy resistance, such as resistance to Sunitinib, the first-line treatment of RCC [15, 16].

We first applied the MTT assay to examine the RCC cells viability in response to Sunitinib treatment. As shown in Fig. 5J, and consistent with the previous report [17], Sunitinib is less effective in suppressing RCC cell viability under hypoxia than under normoxia, likely as a result of increased stem cell formation under hypoxia. Indeed, knocking down lncTCFL5-2 can abolish this difference in the efficacy of Sunitinib between normoxia and hypoxia in RCC cells, consistent with the role of lncTCFL5-2 in enhancing CSCs formation and acting as a critical contributor for the drug sensitivity in RCC cells likely through regulation of CSC formation (Fig. 5J).

To explore the potential therapeutic application of suppressing lncTCFL5-2 in enhancing the current RCC targeted therapy, we examined the effect of a CDK7 inhibitor in this process. CDK7 inhibitors, such as THZ1, have been shown to repress CSCs formation as an inhibitor of transcriptional activation such as the activity of super enhancers [18, 19]. Results revealed that THZ1 could more suppress the expression the lncTCFL5-2 under hypoxia than under normoxia (Fig. 5K). Consistent with the function of lncTCFL5-2 in CSC formation, THZ1 could mimic the effect of knocked-down lncTCFL5-2, thus Sunitinib exhibited similar efficacy in cells both under normoxia and hypoxia while a simultaneous knock down of the lncRNA will further enhance the efficacy of Sunitinib (Fig. 5L). To further implicate the functional significance of lncTCFL5-2 and YBX1 interaction in regulating the sensitivity towards Sunitinib, we found that exogenous expression of the wildtype lncTCFL5-2 can partially reverse the reduction of cell viability in response to Sunitinib and THZ1, while mutant lncTCFL5-2 was even lower than the control, suggesting that THZ1 can suppress the lncTCFL5-2 expression likely through suppressing super enhancer [18] activity to resensitize RCC cells to Sunitinib under hypoxia in RCC cells (Fig. 5M).

Together, results from Fig. 5J–M suggest that lncTCFL5-2 and CSC formation may play critical roles to influence the efficacy of the RCC chemotherapy and THZ1 application may potentially result in significant enhancement of the current RCC targeted therapy by decreasing lncTCFL5-2 expression and CSC formation.

### Clinical significance of AR/lncTCFL5-2/YBX1/SOX2 signaling axis

**Fig. 6:**
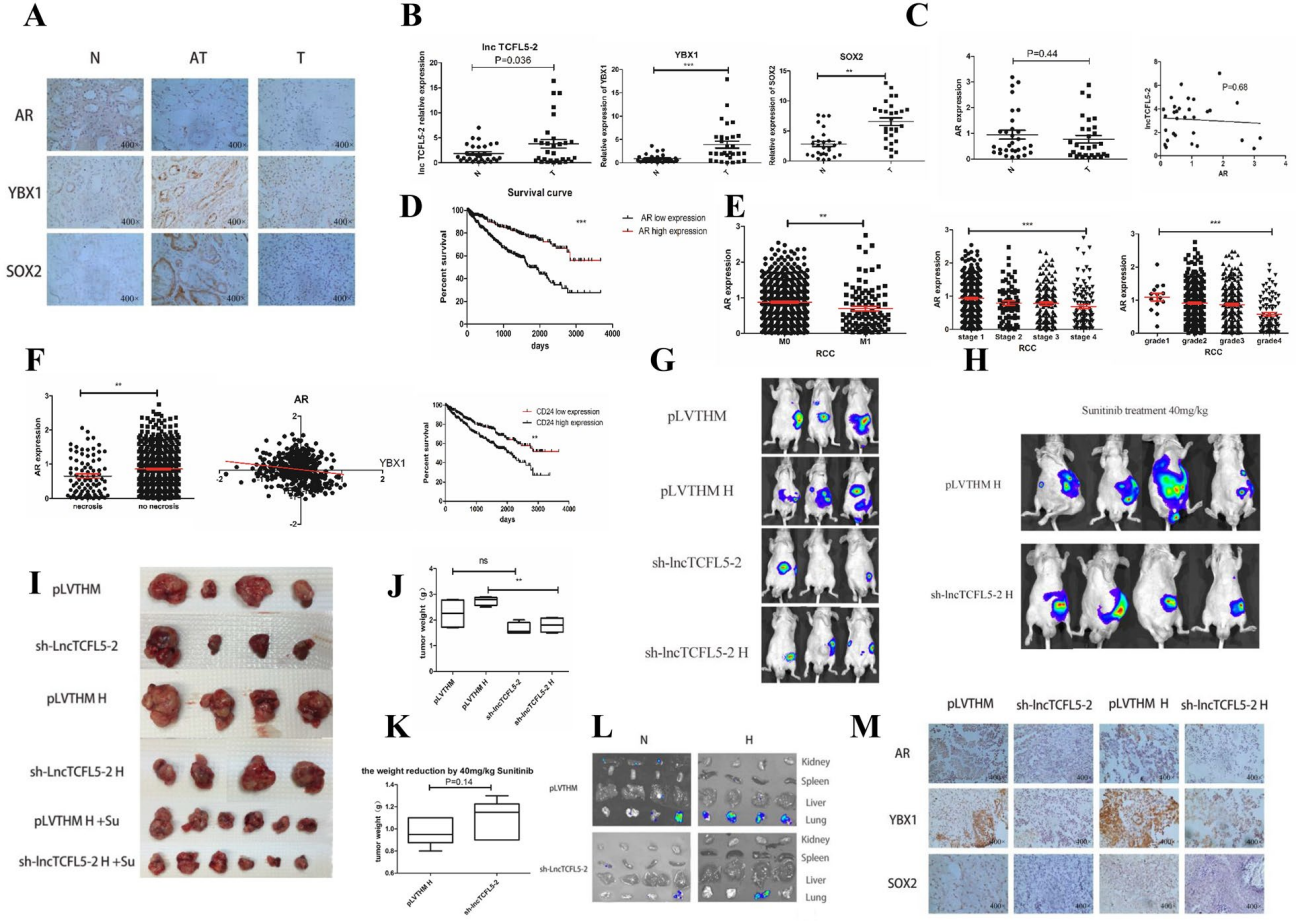
Clinical data and xenograft model demonstrate AR/lncTCFL5-2/YBX1/SOX2/CSCs pathway. **A** IHC staining for AR, YBX1, and SOX2 of tumor (T) tissues, adjacent tumor (AT) tissues and normal (N) tissues in ccRCC samples. **B** Real-time RT-PCR assays for detecting lncTCFL5-2, YBX1 and SOX2 mRNA expressions in tumor and normal tissues. **C** Real-time RT-PCR assays for detecting AR mRNA expression in tumor tissues and normal tissues and the correlation analysis of AR and lncTCFL5-2. **D** 560 RCC patients from TCGA dataset were divided into two groups according to AR expression. Survival curves showed that patients who have a higher AR expression have a higher survival rate. **E** AR expression in M0 and M1 patients, in different grades and different stages. **F** AR expression in RCC sample with necrosis or no necrosis (left panel), the correlation of YBX1 and AR protein (middle panel). 560 RCC patients from TCGA dataset were divided into two groups according to CD24 expression. Survival curves showed that patients who have a higher CD24 expression have a lower survival rate (right panel). **G-M** Results from in vivo mouse studies. **G** IVIS imaging was used to determine the tumor sizes and metastases. **H** We treated mice with 40 mg/kg Sunitinib daily IP for two weeks, IVIS imaging was used to determine the tumor sizes and metastases. **I** After sacrificing the mice the tumor tissues were obtained as shown (N: normoxia, H: hypoxia). **J** Weights of the xenografts were shown in four groups. **K** Weights reduction of the xenografts were shown in two groups after Sunitinib treatment (weight reduction = mean weight treated without Sunitinib-weight treated with Sunitinib). **L** Representative bioluminescent images of different organ metastasis. (N: normoxia, H: hypoxia) **M** The IHC for AR, YBX1, SOX2 of xenografts in four groups (N: normoxia, H: hypoxia)

To link the above in vitro results in human RCC formation and progression, we examined the clinical significance of the AR/lncTCFL5-2/YBX1/SOX2 signaling axis in RCC patient samples. We first used IHC to detect AR, YBX1 and SOX2 protein level in 30 ccRCC patient samples, and the results indicated that AR expression is higher in normal tissues compared to tumor tissues and adjacent tumor tissues, while YBX1 and SOX2 expression is higher in tumor tissues and adjacent tumor tissues compared to normal tissues (Fig. 6A) which is consistent with TCGA database (Additional file 1: Fig. S4A). Consistent with the in vitro cell lines results, expression of lncTCFL5-2 is higher in RCC tissues than in the normal tissues as well as YBX1 and SOX2 (Fig. 6B), and there is a lower *AR* expression in RCC tissues compared to the normal tissues (Fig. 6C), consistent with a negative correlation trend between AR and lncTCFL5-2 and a positive correlation trend between SOX2 and YBX1 or lncTCFL5-2 (Fig. 6C, Additional file 1: Fig. S4B).

We also performed a similar analysis with the TCGA data set and consistent with a previous analysis [7], patients who had a higher AR expression have a higher survival rate (Fig. 6D). Patients who had metastases had a lower AR level on aggregate (Fig. 6E). With an increase of ccRCC stage and grade, there is a corresponding decrease of AR level (Fig. 6E). Furthermore, patients who had a necrosis in tumors, an indicator of hypoxia, have a lower AR expression (Fig. 6F left panel). In addition, there is a statistically significant negative correlation between AR and YBX1 in protein expression (Fig. 6F middle panel). And the CSC marker CD24 is also negatively correlated with patient overall survival (Fig. 6F right panel).

Together, these clinical results from Fig. 6A–F and Additional file 1: S4A-B are consistent role of AR/lncRNA TCFL5-2/YBX1/SOX2 signaling axis found through in vitro studies, and further demonstrated the clinical significance of this signaling axis for the patient prognosis likely as a result of regulating CSC formation.

### The AR/lncTCFL5-2/YBX1/SOX2 signaling axis in RCC xenograft model

In order to test the validity of the in vitro data, we performed the in vivo orthotopic mouse tumor formation assay with the OSRC-2 cells expressing firefly luciferase. The cells were divided into 4 groups, vector control with or without hypoxia for 48 h, and sh-lncTCFL5-2 group with and without hypoxia for 48 h. These cells were inoculated into the left kidney capsule of nude mice and tumor sizes and metastases were evaluated. Once the tumor formation was detectable, we also compared the efficacy of Sunitinib treatment in the groups with and without lncTCFL5-2. The In Vivo Imaging Systems (IVIS) was used to monitor tumor growth. After 8 weeks, we sacrificed the mice and examined the tumor metastasis in lung, liver, spleen, and right kidney with the help of IVIS.

The results showed that RCC cells with a reduction of lncTCFL5-2 pre-cultured under hypoxia and normoxia resulted in significantly slower tumor growth than the control group (Fig. 6G). When mice were treated with 40 mg/kg Sunitinib for two weeks, the tumors were significantly smaller in the sh-lncTCFL5-2 group than the control group (Fig. 6H). After 8 weeks, we sacrificed the mice, and the tumor volumes and weight measurement also confirmed the conclusion that sh-lncTCFL5-2 suppresses tumorigenicity under both normoxia and hypoxia (Fig. 6I, J). In addition, treating mice with 40 mg/kg Sunitinib every day for 2 weeks, the tumor size reduction is more significant in cells with a reduction of lncTCFL5-2 group than the control group (Fig. 6I, K). The tumor metastasis was also evaluated via the IVIS, which revealed that less metastasis occurred in the sh-TCFL5-2 group under both normoxia and hypoxia (Fig. 6L). The IHC staining also indicated that AR expression decreased clearly under hypoxia, and both YBX1 and SOX2 expression decreased sharply in sh-lncTCFL5-2 group consistent with the in vitro finding (Fig. 6M).

**Fig. 7:**
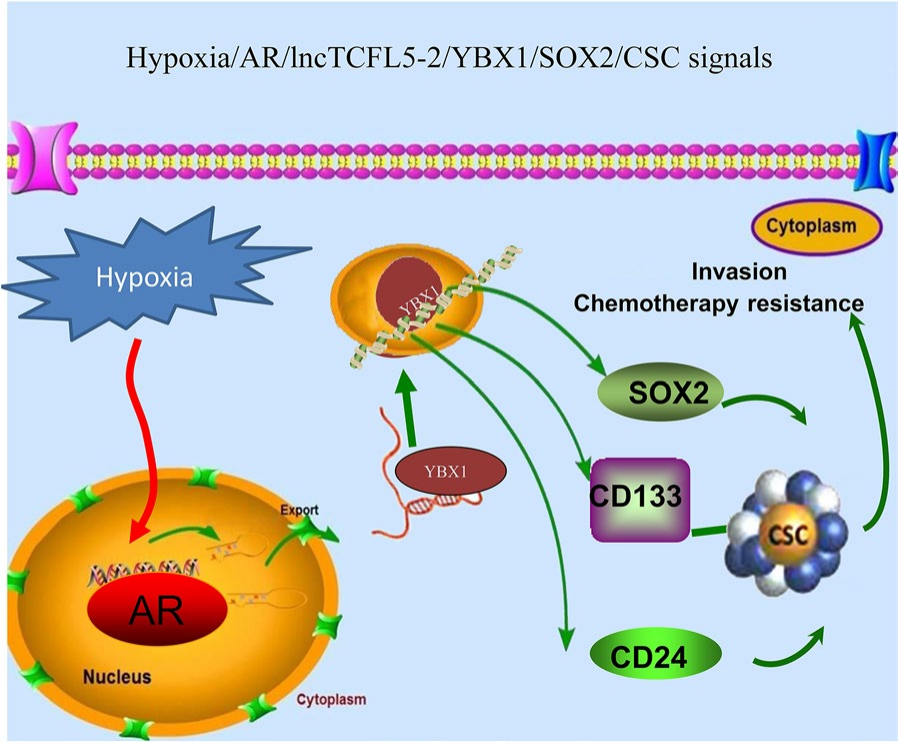
Mechanisms and regulatory pathways of hypoxia increases RCC CSCs phenotype in RCC. Hypoxia may increase RCC CSCs phenotype via altering the AR/lncTCFL5-2/YBX1/SOX2 signaling axis

Together, these data indicated that AR/lncTCFL5-2/YBX1/SOX2 signaling axis plays a critical role in regulating CSC formation in vivo. Repression of lncTCFL5-2 expression likely will promote Sunitinib efficacy for RCC in vivo (Fig. 7).

## Discussion

The clear gender bias of RCC incidence implicates the androgen/AR signaling as a likely promoting factor for RCC initiation and development. Indeed, recent studies supported this view with various cell line studies in vitro and xenograft studies in vivo [20]. However, AR staining in RCC tumors suggested that AR is expressed lower in RCC tumors as compared to normal kidney cells, which indirectly suggests that AR may play a negative role for the RCC progression [7, 21]. These contrasting findings suggest that it is difficult to use AR expression, and not AR function, in any single condition to conclude a role of AR in RCC initiation and progression. The current study indicated that AR might play roles differently in different pathophysiological conditions such as hypoxia, which influences the tumor microenvironment including tumor cells as well as stromal, endothelial, and immune cells [22]. This finding that a single protein can influence differential biological outcomes in different conditions is certainly not new as opposite functions of Ras and Myc have also been found in cancerous cells vs. normal cells [23]. Indeed, AR itself has been found to promote prostate cancer growth yet to suppress prostate cancer metastasis, two seemingly opposite characteristics associated with tumor progression [24]. These results suggest that the overall biological function of a particular protein is largely dictated by the cellular and tissue context, thus targeted therapies based on the functions of these proteins need to be implemented with the understanding of these nuances.

Recent whole transcriptome sequencing has revealed a large number of putative lncRNAs that seem to be involved in a variety of biological processes, including cell-cycle regulation [25], embryonic stem cell (ESC) pluripotency [26, 27], and cancer progression [28]. We provided a first example of a lncRNA that is critical for the hypoxia-regulated RCC stem cells phenotype with the identification of the lncTCFL5-2. Mechanistic studies indicated that this lncRNA can interact through YBX1 to regulate SOX2 expression that in turn regulates the CSCs phenotype as indicated by the sphere formation assay as well as CSCs biomarkers expression. Interestingly, although hypoxia-inducible factors (HIFs) are key transcriptional modulators that mediate the hypoxia signaling associated with multiple processes in RCC progression [29], we found that lncTCFL5-2 is not regulated by HIFs. As shown in the Additional file 1: Fig. S4C, a reduction of HIF2a through shRNA-mediated knock down or a small molecule inhibitor in both SW839 and OSRC-2 cells failed to alter the lncTCFL5-2 expression in response to hypoxia. It is clear that AR is important for this regulation, but whether AR serves as a sensor of oxygen availability or as a cofactor together with other oxygen sensors such as the JmjC family of histone demethylases [30] to regulate lncRNA-TCFL5-2 expression remains to be determined.

YBX1 is a DNA/RNA-binding protein that can bind to the promoter sequence containing an inverted CCAAT box (Y-box sequence) and regulate transcription as well as translation [31]. YBX-1 is fundamentally cytoplasmic in normal cells, and nuclear in transformed cells, or after specific stimuli. Many inducible transcription factors (TFs) are found in the cytoplasm or membrane bound, and are transferred to the nucleus only after a specific stimulus [31]. Our results from RIP assays revealed that lncTCFL5-2 indeed interacted with YBX1 compared to AR that failed to interact with this lncRNA. Therefore it is likely that lncTCFL5-2/YBX1 complex translocates from the cytoplasm to the nucleus to regulate SOX2 transcription.

The relationship of YBX1 and SOX2 has been reported in several studies [14, 32] with opposite directions in different tissues. Fotovati A et al. [32] reported a positive correlation between YBX1 and SOX2 in glioma cells, while a negative relationship was found in breast cancer cells [14]. In our study, we found lncTCFL5-2 can interact with YBX1 and positively regulate SOX2 at both mRNA and protein levels. The specificity of this interaction is underscored by the activity of the mutant lncTCFL5-2 in 864-936nt region, which failed to interact with YBX1 while simultaneously failed to reverse the effect of AR-suppressed CSCs phenotypes for marker gene expressions and sphere forming abilities. On the other hand, it is not possible to exclude the possibility that this mutation affects other unknown functions of lncTCFL5-2 through which affects the phenotype that we observed.

It is worth noting that the expression of YBX1 and SOX2 in tissues adjacent to tumor area is significantly higher than that in normal tissues. It is possible that the microenvironment surrounding tumors even though phologically normal also undergoes metabolic changes during tumor growth such as when under hypoxia. These issues require further investigation to be clarified.

Currently the effective treatments for advanced RCC include those that target VEGF and mTOR signaling. However, resistance to these therapies invariably develops in patients. Understanding the mechanisms that contribute to the target therapy resistance is urgently needed to provide survival benefits for RCC patients. The CSCs phenotype is closely associated with the therapy resistance likely due to the metabolic adaptation of CSCs to the drug treatment [33]. Our finding that a lncRNA is critical for the CSC phenotype, as well as the RCC sensitivity towards the first line therapy Sunitinib, is an important advance towards rationally designing more effective therapies for advanced RCC. In particular, we found that a CDK7 inhibitor, which is currently used in clinical trial, can effectively repress the expression of lncTCLF5-2, and enhance the efficacy of Sunitinib for RCC, further substantiating the notion that this lncRNA is critical for the CSCs phenotype as well as resistance to targeted therapy. CDK7 is a member of cyclin-dependent kinases family (CDKs) which have critical roles in transcription initiation and elongation [18]. Inhibition of transcriptional CDK7 primarily affects the accumulation of transcripts with short half-lives, including anti-apoptosis family members and cell cycle regulators [34]. It was also demonstrated that CDK7 is a positive regulator of super enhancer-mediated transcription [19]. Our finding that the CDK7 inhibitor can effectively repress the expression of lncTCLF5-2 suggests that lncTCFL5-2 might be also under the control of a super enhancer. These findings established the foundation to search for more effective means to suppress the lncTCFL5-2 expression and CSCs formation to enhance efficacy for RCC therapy.

In conclusion, we demonstrated that hypoxia decreased the expression of AR, which plays a critical role of regulating CSCs formation through lncTCFL5-2, which in turn regulates the SOX2 level through YBX1. Targeting this newly identified signal may help physicians to improve the targeted therapy with Sunitinib to better suppress the RCC progression.

## Conclusions

These findings suggest that hypoxia may increase the RCC CSC phenotype via altering the AR/lncTCFL5-2/YBX1/SOX2 signaling axis and a potential therapy to target this newly identified signal perhaps may help improve the targeted therapy with Sunitinib to better suppress RCC progression.

## Supplementary Information

**Additional file 1: Figure S1. The expression of protein level related to AR. ** (A) The AR protein level expression under hypoxia (H) and normoxia (N) in OSRC-2 and SW839 cells. (B) The EZH2 and SRC expression under hypoxia (H) and normoxia (N) in OSRC-2 cells. (C) The efficiency of shRNA-AR at the protein level (left) in SW839 cells and the efficiency of oe-AR in OSRC-2 cells (right) determined at mRNA level. (D) The HIF1α expression under hypoxia (H) and normoxia (N) in OSRC-2 cells. **Figure S2.** **The** **supplement figures demonstrated how to focus on lncTCFL5-2.** (A) The fold change of lncRNAs in microarray analysis of SW839 cell inresponse to hypoxia. (B) The list of the top 20 downregulated lncRNAs by hypoxia. (C) SW839 and OSRC-2 cells were lentivirally transduced with sh-AR and oe-AR, respectively, and then cells were exposed to hypoxia or normoxia for 2 days. Total RNAs were analyzed by Q-PCR for the 20 down regulated lncRNAs. (D) OSRC-2 cells were virally transduced with oe-AR and pWPI, and then cells exposed to hypoxia (H) or normoxia (N) for 2 days. Q-PCR was used to show 3 lncRNAs expressions. The lncRNA expressions were calculated by hypoxia/normoxia. (E) SW839 and OSRC-2 cells were lentivirally transduced with sh-lncTCFL5-2 sequence 1 and sh-lncTCFL5-2 sequence 2, then cells were exposed to hypoxia or normoxia for 2 days. Sphere formation assay was used to demonstrate the CSCs number. (F) RCC cells were lentivirally transduced with sh-lncTCFL5-2 or oe-lncTCFL5-2 then exposed to hypoxia and normoxia for 2 days. AR expression was evaluated by qPCR and Western-blot. **Figure S3.** **The supplement figures demonstrated** **AR/lncTCFL5-2/YBX1/SOX2 signaling axis.** (A) ACHN cells were lentivirally transduced with oe-AR and cells exposed to hypoxia or normoxia for 2 days, qPCR analysis of the expression of lncTCFL5-2. (B) OSRC-2 cells were treated with the anti-androgen enzalutamide (Enz) (10 μM) for 48h, qPCR was used to test the expression of CSCs biomarkers, including CD24, CD133, PAX2, SOX2 and CD105. (C) The AR and YBX1 protein levels were tested in immunoprecipitates with anti-AR and anti-YBX1 antibodies in 293T cells. (D) The lncTCFL5-2 can be detected in the immunoprecipitate with anti-YBX1 at endogenous level in OSRC-2 cells. (E) OSRC-2 cells were lentivirally transduced with oe-AR or oe-AR oe-mutant lncTCFL5-2 or oe-AR oe-lncTCFL5-2 and cells exposed to hypoxia for 2 days, qPCR analysis of the expressions of AR and lncTCFL5-2. (F) OSRC-2 cells were exposed to hypoxia and normoxia for 2 days. Then immunofluorescent staining was used to detect the CD24 and CD133 double-stained cells. **Figure S4.** **The relationship of AR, YBX1, lncTCFL5-2, and SOX2.** (A) The AR, YBX1, and SOX2 expression in ccRCC tumor tissues and normal tissues Based on mining TCGA Data. (B) Real-time RT-PCR assays for detecting the correlation analysis of SOX2 and YBX1 or lncTCFL5-2. (C) SW839 and OSRC-2 cells were lentivirally transduced with sh-HIF2α, or treated with a small molecule inhibitor and then exposed to hypoxia or normoxia for 2 days. Total RNA was analyzed for expression of lncTCFL5-2 by real-time PCR.

## Data Availability

The authors declare that all data and materials are available on request.

## Abbreviations

RCC: Renal cell carcinoma
AR: Androgen receptor
CSC: Cancer stem cells
AREs: AR-response-elements
YBX1Es: YBX1-response-elements
VEGF: Vascular endothelial growth factor
mTOR: Mammalian target of rapamycin
VHL: Von Hippel-Lindau
HIF: Hypoxia inducible factor
qRT-PCR: Real-time quantitative polymerase chain reaction
RIP: RNA immunoprecipitation
RNA-seq: RNA sequencing
TCGA: The Cancer Genome Atlas
ChIP: Chromatin Immunoprecipitation Assay
ESC: Embryonic stem cell
