# Supplementary material for: Hypoxia increases RCC stem cell phenotype via altering the androgen receptor (AR)-lncTCFL5-2-YBX1-SOX2 signaling axis
Source: Cell Biosci. 2022 Nov 17;12:185. doi: 10.1186/s13578-022-00912-5 (PMC9670551; doi:10.1186/s13578-022-00912-5)
Supplement: Supplementary file 1 — Additional file 1: Figure S1. The expression of protein level related to AR. (A) The AR protein level expression under hypoxia (H) and normoxia (N) in OSRC-2 and SW839 cells. (B) The EZH2 and SRC expression under hypoxia (H) and normoxia (N) in OSRC-2 cells. (C) The efficiency of shRNA-AR at the protein level (left) in SW839 cells and the efficiency of oe-AR in OSRC-2 cells (right) determined at mRNA level. (D) The HIF1α expression under hypoxia (H) and normoxia (N) in OSRC-2 cells. Figure S2. The supplement figures demonstrated how to focus on lncTCFL5-2. (A) The fold change of lncRNAs in microarray analysis of SW839 cell inresponse to hypoxia. (B) The list of the top 20 downregulated lncRNAs by hypoxia. (C) SW839 and OSRC-2 cells were lentivirally transduced with sh-AR and oe-AR, respectively, and then cells were exposed to hypoxia or normoxia for 2 days. Total RNAs were analyzed by Q-PCR for the 20 down regulated lncRNAs. (D) OSRC-2 cells were virally transduced with oe-AR and pWPI, and then cells exposed to hypoxia (H) or normoxia (N) for 2 days. Q-PCR was used to show 3 lncRNAs expressions. The lncRNA expressions were calculated by hypoxia/normoxia. (E) SW839 and OSRC-2 cells were lentivirally transduced with sh-lncTCFL5-2 sequence 1 and sh-lncTCFL5-2 sequence 2, then cells were exposed to hypoxia or normoxia for 2 days. Sphere formation assay was used to demonstrate the CSCs number. (F) RCC cells were lentivirally transduced with sh-lncTCFL5-2 or oe-lncTCFL5-2 then exposed to hypoxia and normoxia for 2 days. AR expression was evaluated by qPCR and Western-blot. Figure S3. The supplement figures demonstrated AR/lncTCFL5-2/YBX1/SOX2 signaling axis. (A) ACHN cells were lentivirally transduced with oe-AR and cells exposed to hypoxia or normoxia for 2 days, qPCR analysis of the expression of lncTCFL5-2. (B) OSRC-2 cells were treated with the anti-androgen enzalutamide (Enz) (10 μM) for 48h, qPCR was used to test the expression of CSCs biomarkers, including CD24, CD [file 13578_2022_912_MOESM1_ESM.pdf]

Supplement Figure 1

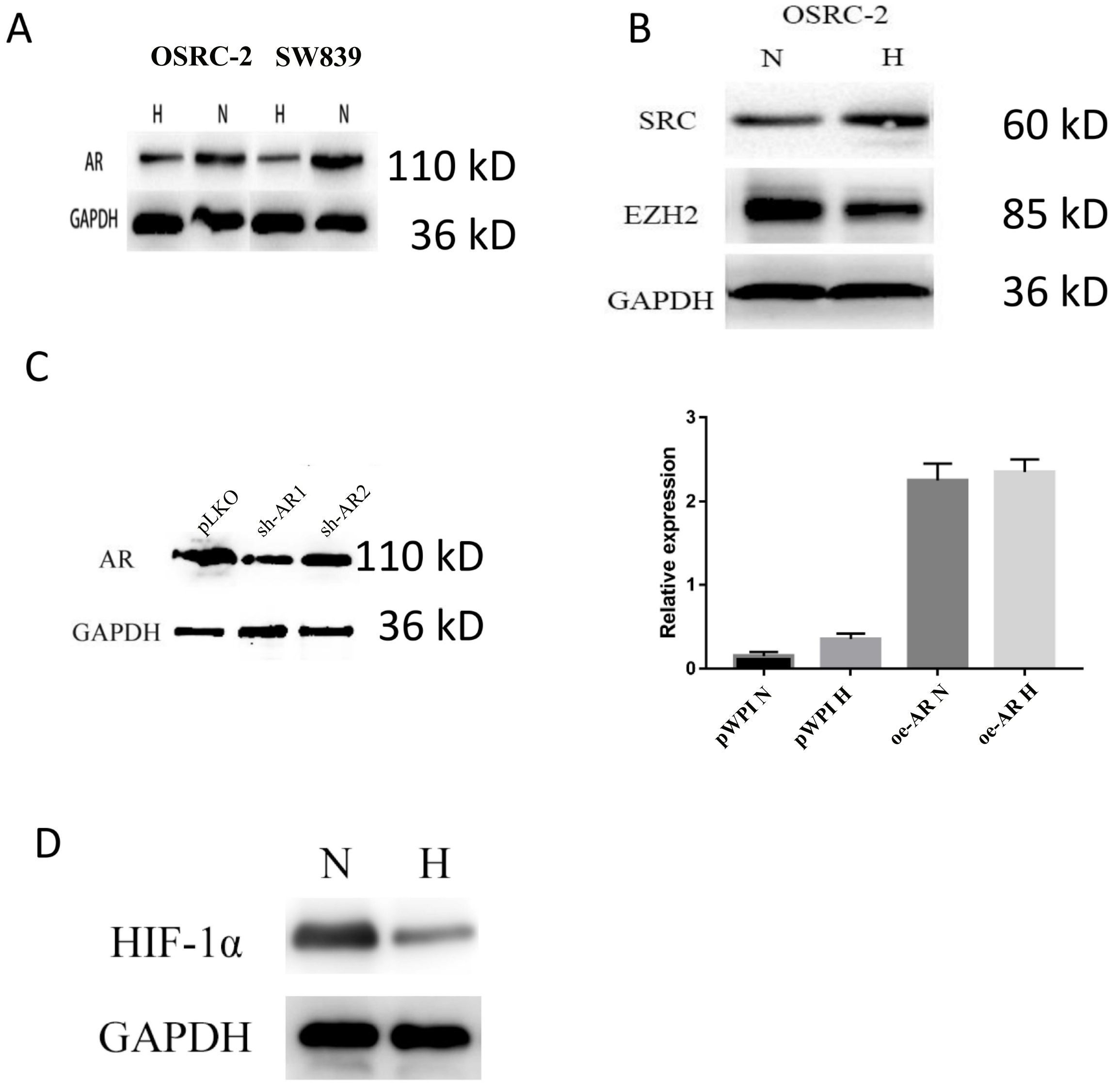

Supplement Figure 2

A

LncRNA microarray analysis of SW839 in response to hypoxia

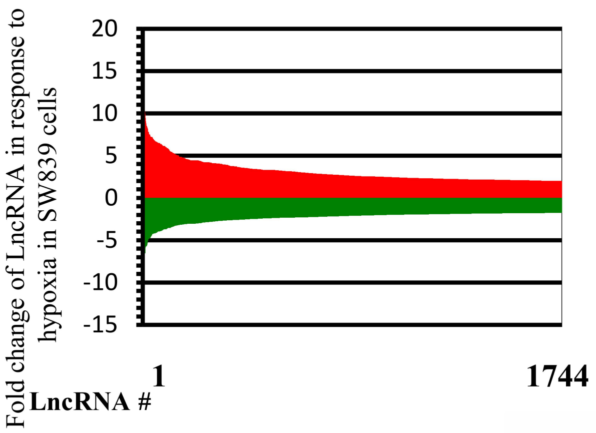

B

Down regulate

XLOC_010642	-9.1271263
CTD-2501M5.1	-7.5389561
CTD-2194A8.2	-7.5129366
RP11-328C8.5	-6.7631405
RNF157-AS1	-6.6016546
CTD-3023L14.2	-6.5963231
RP11-115N4.1	-6.556549
RP11-143N13.2	-6.5215188
CTD-2501M5.1	-5.7568959
XLOC_006291	-5.7404252
RP11-389G6.3	-5.6612504
CTD-2194A8.2	-4.9825711
CTC-454M9.1	-4.8427792
XLOC_008630	-4.7648615
XLOC_011210	-4.7567543
RP11-341G23.4	-4.7432455
XXbac-BPG308K3.5	-4.3845441
RP11-163N6.2	-4.3783887
CTD-3023L14.1	-4.2403057
RP11-341G23.4	-4.1617539

-10 0

C

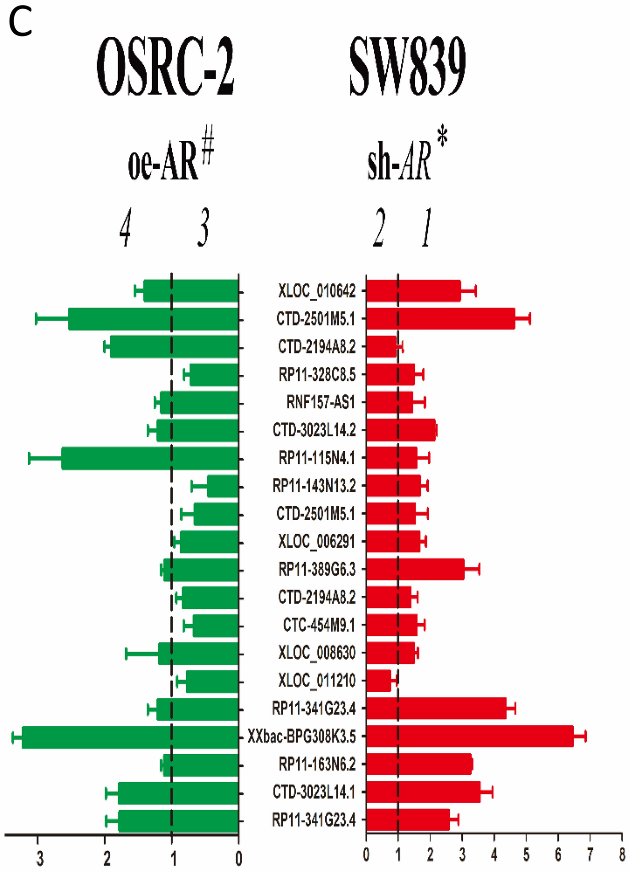

*fold of repression of hypoxia vs normoxia in sh-AR/that in control cells

fold of repression of hypoxia vs normoxia in oe-AR/that in control cells

1,3：AR suppresses lncRNA induction by hypoxia；

2,4：AR promotes lncRNA induction by hypoxia.

D

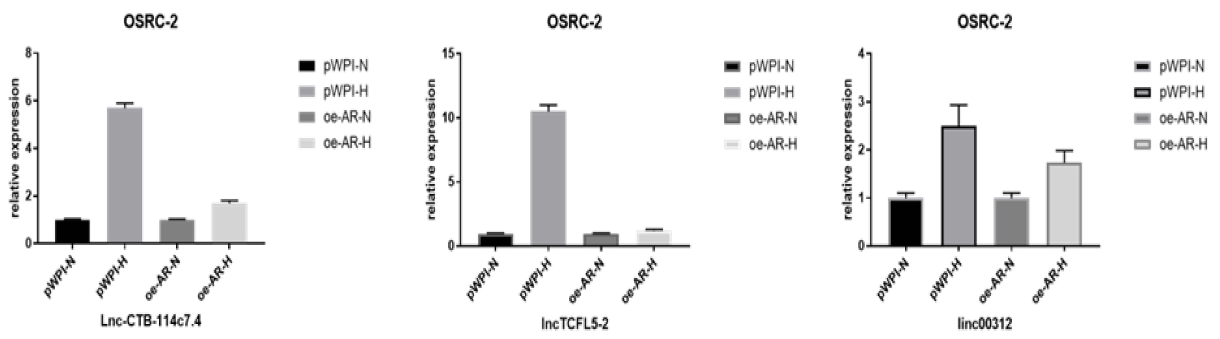

Supplement Figure 2

E

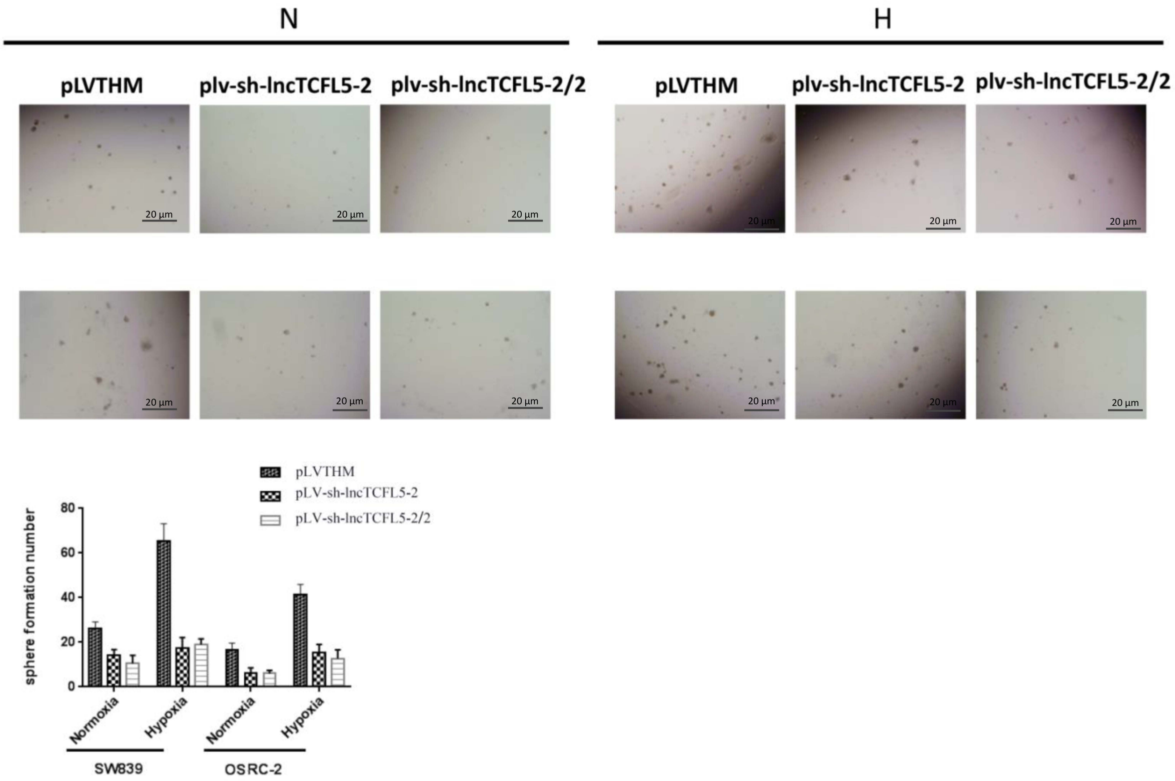

F

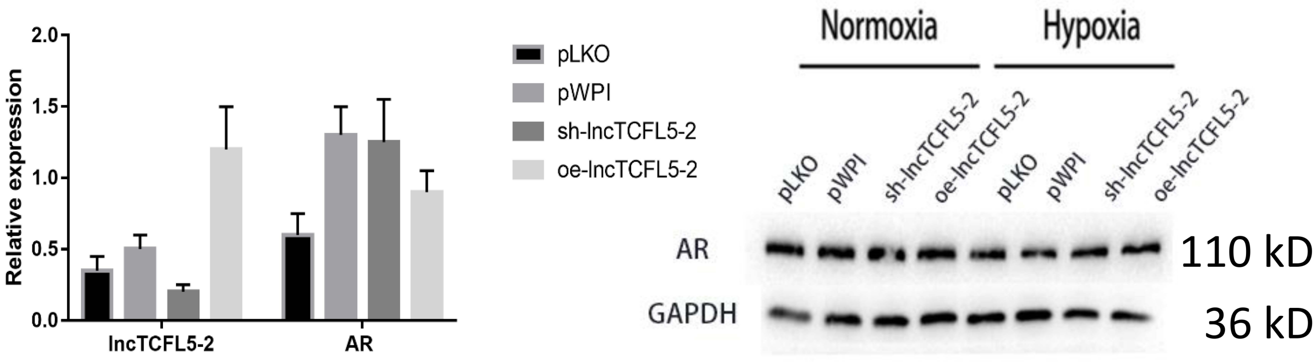

Supplement Fiugre 3

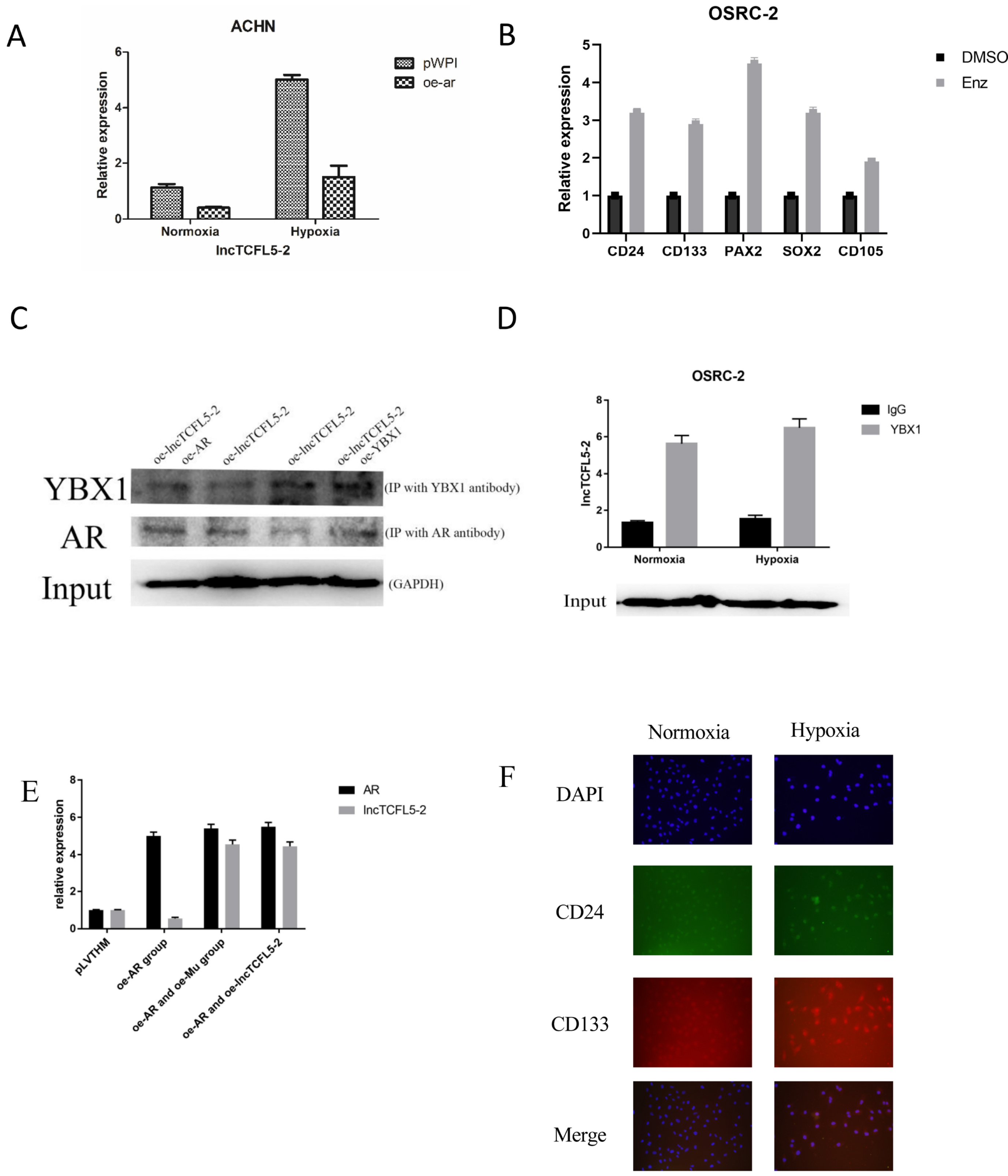

Supplement Figure 4

A

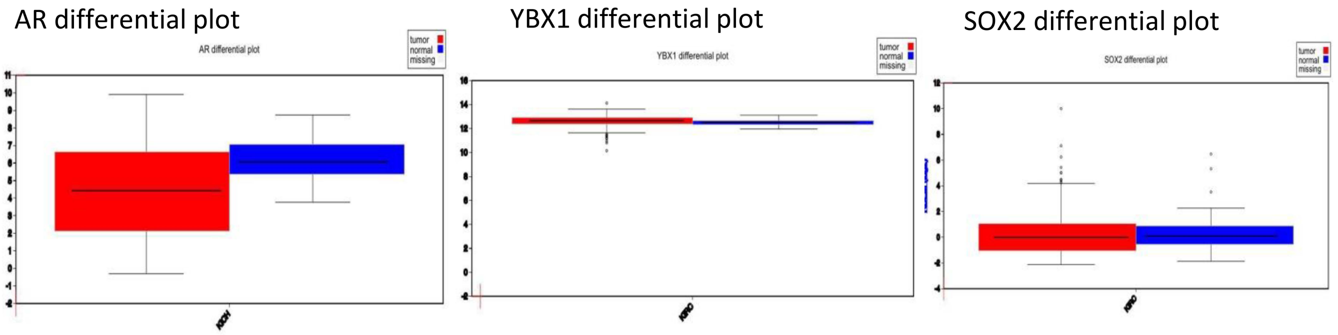

B

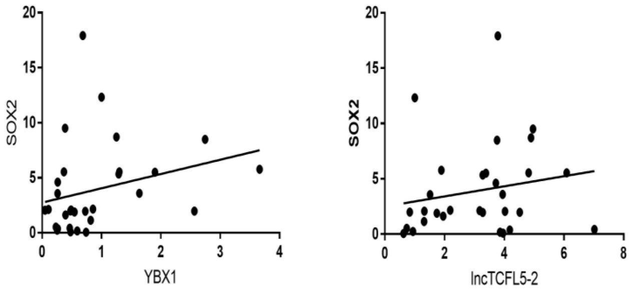

C

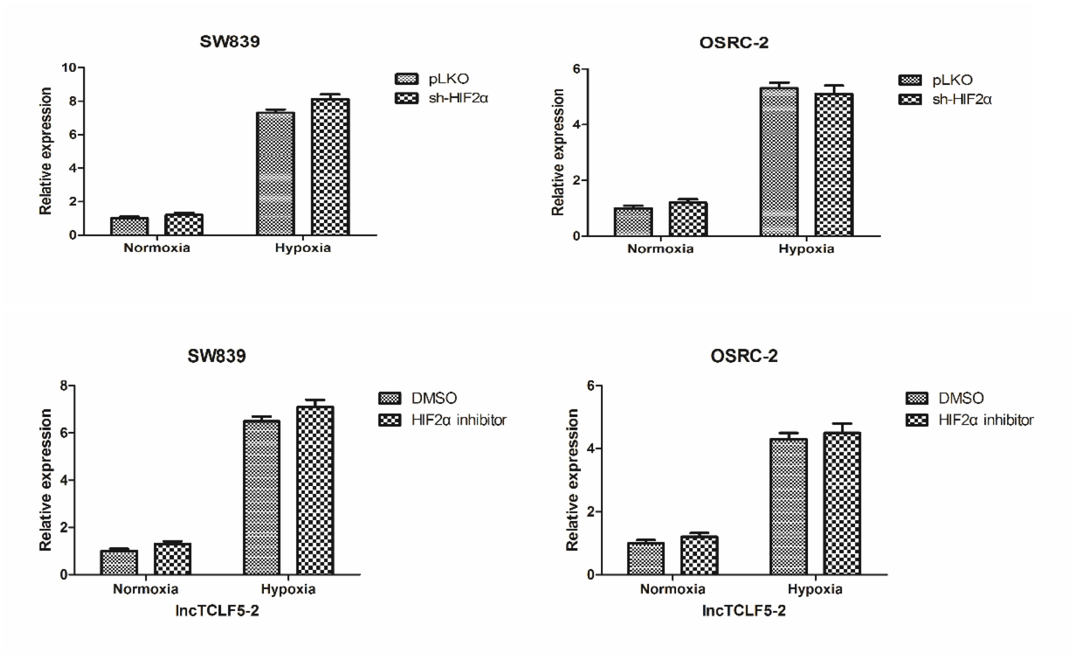

Primers used for vectors construction and PCR	
**Primer names**	**Sequences**
YBX1	F: 5'AGACAGTAGGGGTCGGGAGT3'
	R: 5'TCAGGTCCACTGCACTCTTG3'
SOX2	F: 5'GCCGAGTGGAAACTTTTGTCG3'
	R: 5'GGCAGCGTGTACTTATCCTTCT3'
NANOG	F: 5'AAGGTCCCGGTCAAGAAACAG3'
	R: 5'CTTCTGCGTCACACCATTGC3'
OCT4	F: 5'CTGGGTTGATCCTCGGACCT3'
	R: 5'CATCGGAGTTGCTCTCCAC3'
CD24	F: 5'GCAGAATGTGGACATGAAGA'
	R: 5'ATGCTAAAAAAGATTCGCAATG3'
CD133	F: 5'ATCAGAACTGCAATCTGCACA3'
	R: 5'AGAAGATCCCTGTCACAATTCC3'
AR	F: 5'CAGGAGGAAGGAGAAAAC3'
	R: 5'GATAACAAGGCAGCAAAG3'
RP1-27K12.4	F: 5'GCAAAAGCTCTCACCACACA3'
	R: 5'CTTAGGCTCTTGGGTCATCG3'
CTB-114C7.4	F: 5'AAGTCAGACCCCAACACGTC3'
	R: 5'AGAGGCCAGTGTAGCTGGAA3'
WDR13	F: 5'TTCCACCATGAACTCCATCA3'
	R: 5'AGCAGCAGGATGGTCTGAGT3'
SSBP1	F: 5'GCGATCAGGGGATAGTGAAG3'
	R: 5'TTGCTTGTCGCCTCACATTA3'
lncTCFL5-2	F: 5'CTCATCCAGACAGCTGGACA3'
	R: 5'AAGCGGCTCTATGGAAGACA3'
ENST00000419440	F: 5'TTCCAGCATGGAAGTCAGTG3'
	R: 5'GAGTCAGGGGCTCAGAGTTG3'
ENST00000576489	F: 5'GAAGAACTGTTGCCCTCTGC3'
	R: 5'CCCCTGCTTCTCTACCACTG3'
ENST00000419440	F: 5'TTCCAGCATGGAAGTCAGTG3'
	R: 5'GAGTCAGGGGCTCAGAGTTG3'

SHMT2	F: 5'AAGAGTCACGCGCTTTCAAT3'
	R: 5'GTAGGGGTGCTCACTCTG GA3'
RP11-193H5.1	F: 5'GTGCTGCCAATCACTGAAGA3'
	R: 5'CTCTGCTGATCCCACACTCA3'
RP11-907D1.2	F: 5'GTGCTGCCAATCACTGAAGA3'
	R: 5'CTCTGCTGATCCCACACTCA3'
RP11-193H5.1	F: 5'GTGCTGCCAATCACTGAAGA3'
	R: 5'CTCTGCTGATCCCACACTCA3'
LINC0031	F: 5'ATGGCTGTTGGTCATTCACA3'
	R: 5'CCAAAGGAATCAGACCAGGA3'
CTD-2066L21.3	F: 5'GTGCTGCCAATCACTGAAGA3'
	R: 5'CTCTGCTGATCCCACACTCA3'
XLOC_009326	F: 5'GCCAGCTGGAAGAGTATTCG3'
	R: 5'TTTGCTTCCAACAGCAACTG3'
RP11-63M22.2	F: 5'CACATTTCCAGCCTCTCACA3'
	R: 5'CACAGCTGAAGAAGGGAAGG3'
FAM25D	F: 5'AAGTGGTGAAGGAGGTGGTG3'
	R: 5'CCAAGTTTGCCCAGACTCTC3'
RP11-193H5.1	F: 5'GTGCTGCCAATCACTGAAGA3'
	R: 5'CTCTGCTGATCCCACACTCA3'
XLOC_008710	F: 5'AAACCATCACATTCGGCTTC3'
	R: 5'TTGTTTTGCCACTTCTGCTG3'
XLOC_002534	F: 5'CTCTGAAGTGGATGGGAAGC3'
	R: 5'GATCAGGAAACAGGCCAGAC3'
RP11-221N13.3	F: 5'CCTGAAAAGGAGCCAGAGTG3'
	R: 5'GACGAGAAACTTCCCCAACA3'
RP11-534G20.3	F: 5'CCCTACACCAAAGTGGGAGA3'
	R: 5'TTATTCTTTCACCGGCCAAG3'
RP11-513I15.6	F: 5'CACCCATGAGAAGGAGGTGT3'
	R: 5'AGCCTTGCTTGGTACTGCAT3'
XLOC_010642	F: 5'TGGGGATCTTCAGTGTCTCA3'
	R: 5'GCACATTCGATCTTCAAGTGAC3'

RP11-534G20.3	F: 5'CCCTACACCAAAGTGGGAGA3'
	R: 5'TTATTCTTTCACCGGCCAAG3'
CTD-2501M5.1	F: 5'TTCAAATCCGGACCCTACTG3'
	R: 5'GTAGGCTTCTGAGGCGATTG3'
CTD-2194A8.2	F: 5'GGCGATCTACAAACCAAGGA3'
	R: 5'CGATTTTGGAGGCTGAGAAG3'
RP11-328C8.5	F: 5'AAGCTTGTGACTGGGAGGAA3'
	R: 5'GATTCCGGTTTTGAAGACGA3'
CTD-2194A8.2	F: 5'GGCGATCTACAAACCAAGGA3'
	R: 5'CGATTTTGGAGGCTGAGAAG3'
RNF157-AS1	F: 5'CCTCCTACGTCCTTGCTCTG3'
	R: 5'GGTCAAGGAGACAGGTCCAA3'
CTD-3023L14.2	F: 5'TGCAGCATCACAGTGACAGA3'
	R: 5'TCCTGACCTGGTTGCTCTTT3'
RP11-115N4.1	F: 5'GCTTGGAGATTCCCATTTCA3'
	R: 5'AGCTGGATGCAATACCCTTG3'
RP11-143N13.2	F: 5'GGAGCCGAGGCATATGATAA3'
	R: 5'ACATCAACCCCAAAGACTGC3'
CTD-2501M5.1	F: 5'TTCAAATCCGGACCCTACTG3'
	R: 5'GTAGGCTTCTGAGGCGATTG3'
XLOC_006291	F: 5'CAAGTGAAGGCAACCCAGTT3'
	R: 5'CTAACCTCGGCTCACTCCTG3'
RP11-389G6.3	F: 5'CACGATTTGGGAGGGTATTG3'
	R: 5'AGAGGGGCACAATGAACAAC3'
CTD-2194A8.2	F: 5'GAAGCTGCACACCTTTGACA3'
	R: 5'CGGTTTCTGGAAAGATCCAA3'
CTC-454M9.1	F: 5'CGGATCCTAAGGCAGTTTTG3'
	R: 5'GCTGCAGTGAACCAAGATCA3'
XLOC_008630	F: 5'AGATGTGGCAGAAAGGATGC3'
	R: 5'TGGGACTCAGGCCTCTTCTA3'
XLOC_011210	F: 5'ACACACTGCTTCCCTGCTCT3'
	R: 5'AGCATGAGGGTAGAGGCTGA3'

RP11-341G23.4	F: 5'CACTAGGTGGCATGAGCAGA3'
	R: 5'TCCCAGAATCTCCAAGGCTA3'
XXbac-BPG308K3.5	F: 5'GCGTTTCTCCACCTGTCTTC3'
	R: 5'CCGTCTTTGTCTTCCAGCTC3'
RP11-163N6.2	F: 5'ATGCCTTCACACCACTTTCC3'
	R: 5'GAAGGGACCTTCCTGGTCTC3'
CTD-3023L14.1	F: 5'TCACACCTGTAATCCCAGCA3'
	R: 5'CCAAGTTCTGGATGGGAGAA3'
RP11-341G23.4	F: 5'TCGTGGGTGTGAAGTGACAT3'
	R: 5'CAAGCTGACTGAAGCCAGTG3'
nanog promoter pgl3	F: 5'CTATCGATAGGTACCGAGCTTACAAAACCCTAGTTCTCAT3'
	R: 5'CCAAGCTTACTTAGATCGCAGTTCTTTGCAGA GGG GGTC3'
OCT4 promoter pgl3	F: 5'CTATCGATAGGTACCGAGCTAGGTGACACAAGTAAGGTAA3'
	R: 5'CCAAGCTTACTTAGATCGCAAGGCCGGCGGAATCACGTGC3'
PLVTHM(SalI)-sh-LI	F: 5'CGCGTGCAATAGTGACATGAGCACTTGACTACCCGGTCGACGTAGTCAAGTGCTCATGTCACTATTGCTTTTTG3'
	R: 5'CGCAAAAAGCAATAGTGACATGAGCACTTGACTACGTCGACCGGGTAGTCAAGTGCTCATGTCACTATTGCA3'
sox2-YBX1E mutatio	F: 5'CCACCAGGATCCAAATCTACGGGGAAAAT3'
	R: 5'AGATTTGGATCCTGGTGGCCGCAAAGCCC3'
PLVTM(SalI)-sh-Sox	F: 5'CGCGTACCAAGACGCTCATGAAGAAGGATAACCCGGTCGACGTTATCCTTCTTCATGAGCGTCTTGGTTTTTG3'
	R: 5'CGCAAAAAACCAAGACGCTCATGAAGAAGGATAACGTCGACCGGGTTATCCTTCTTCATGAGCGTCTTGGTA3'
SOX2 Ybx-1ChiP	F: 5'CCCCCTTTCATGCAAAAC3'
	R: 5'GGGGCTGTCAGGGAATAAAT3'
SOX2 Ybx-2ChiP	F: 5'CGCACCTTAGCTGCTTCC3'
	R: 5'AGCAACAGGTCACACCACAC3'
SOX2 Ybx-3ChiP	F: 5'TGAGAGAGTGTTGGCACCTG3'
	R: 5'ATTTCTGGAAACAGCCAGTG3'
SOX2 Ybx-4ChiP	F: 5'ACCTGTGCCTGGAGAGCAT3'
	R: 5'TGTATGGAGGTGGCTTTTGG3'
SOX2 Ybx-5ChiP	F: 5'GGCTTTGTTTGACTCCGTGT3'
	R: 5'CCAGGACCCAAGAGGGTAAT3'
SOX2 Ybx-6ChiP	F: 5'GCAGGAAGGTTGATTGGAAA3'
	R: 5'CTACCAGCCACGTTCCATTT3'

SOX2 Ybx-7ChiP	F: 5'TCTTCCATCCCCCTCTTTTT3'
	R: 5'CCTGCTGGTAGATTCGCTTT3'
lncTCFL5-2 pgl3 gibs	F: 5'CTATCGATAGGTACCGAGCTTAGTCCCTGTGGACCCTCAC3'
	R: 5'CCAAGCTTACTTAGATCGCAGGAGGACCTGGGGAAACAAA3'
ARE-1 TCFL5-2	F: 5'GTGCAATGGAAGGGATGTTT3'
	R: 5'GGGGTCTCAGGTATGAGTGG3'
ARE-2 TCFL5-2	F: 5'CTCCCCTGCCAGGCTATC3'
	R: 5'CATTCATTCCATCCCCGTAA3'
ARE-3 TCFL5-2	F: 5'CACAGCCCCTTAGCACAGAT3'
	R: 5'TCTCACGCTTTTATGCCATC3'
ARE-4 TCFL5-2	F: 5'GGAAGTGTCGGTTCACACCT3'
	R: 5'CCCTCCCACACACACCAC3'
ARE1 sequence	AGTGTCTGGGACAGAAGTGGAATTCC
ARE2 sequence	CACTGGGGAATCCAGGATTGTCCCCCACCTTCA
ARE3 sequence	CCACTGCTGCTGGGACAAATTATAACAAA
ARE4 sequence	TGTTTGGACGGACAGTGTCTCAAAAACCCA

Supplement Figure 1. (A) The AR protein level expression under hypoxia (H) and normoxia (N) in SW839 cells. (B) The EZH2 and SRC expression under hypoxia (H) and normoxia (N) in OSRC-2 cells. (C) The efficiency of shRNA-AR at the protein level and the efficiency of oe-AR determined at mRNA level.(D) The HIF1α expression under hypoxia (H) and normoxia (N) in OSRC-2 cells.

Supplement Figure 2. (A) The fold change of lncRNAs in microarray analysis of SW839 in response to hypoxia. (B) The list of top 20 downregulated lncRNAs by hypoxia. (C) SW839 and OSRC-2 cells were lentivirally transduced with sh-AR and oe-AR, and then cells were exposed to hypoxia or normoxia for 2 days. Total RNAs were analyzed by Q-PCR for the 20 down regulated lncRNAs. (D) OSRC-2 cells were virally transduced with oe-AR and pWPI, and then cells exposed to hypoxia (H) or normoxia (N) for 2 days. Q-PCR was used to show 3 lncRNAs expressions. The lncRNA expressions were calculated by hypoxia/normoxia. (H) OSRC-2 cell were lentivirally transduced with oe-AR then exposed to hypoxia and normoxia for 2 days. Three lncRNAs expression were evaluated by qPCR. (E) SW839 and OSRC-2 cells were lentivirally transduced with sh-lncTCFL5-2 sequence 1 and sh-lncTCFL5-2 sequence 2, then cells were exposed to hypoxia or normoxia for 2 days. Sphere formation assay to demonstrate the CSCs number. (F) RCC cells were lentivirally transduced with sh-lncTCFL5-2 or oe-lncTCFL5-2 then exposed to hypoxia and normoxia for 2 days. AR expression was evaluated by qPCR and Western-blot.

Supplement Figure 3. (A) ACHN cell were lentivirally transduced with oe-AR and cells exposed to hypoxia or normoxia for 2 days, qPCR analysis of the expression of lncTCFL5-2. (B) OSRC-2 cell were treated with the anti-androgen enzalutamide (10 μM) for 48h, qPCR was used to test the expression of CSCs biomarkers, including CD24, CD133, PAX2, SOX2 and CD105. (C) The AR and YBX1 protein levels were tested in immunoprecipitates with anti-AR and anti-YBX1 antibodies. (D) The LncTCFL5-2 can be detected in the immunoprecipitate with anti-YBX1 at endogenous level in OSRC-2 cells. (E) OSRC-2 cell were lentivirally transduced with oe-AR or oe-AR oe -mutant lncTCFL5-2 or oe-AR oe -lncTCFL5-2 and cells exposed to hypoxia for 2 days, qPCR analysis of the expression of AR and lncTCFL5-2. (F) OSRC-2 cell were exposed to hypoxia and normoxia for 2 days. Then immunofluorescent

staining was used to detect the CD24 and CD133 double-stained cells.

Supplement Figure 4. (A) The AR, YBX1,SOX2 expression in ccRCC tumor tissue and normal tissue Based on Mining TCGA Data. (B) Real-time RT-PCR assays for detecting the correlation analysis of SOX2 and YBX1 or lncTCFL5-2.（C）SW839 and OSRC-2 cells were lentivirally transduced with sh-HIF2α, or treated with a small molecule inhibitor and then exposed to hypoxia or normoxia for 2 days. Total RNA was analyzed for expression of lncTCFL5-2 by real-time PCR.
